# Determining Tipping Points and Responses of Macroinvertebrate Traits to Abiotic Factors in Support of River Management

**DOI:** 10.3390/biology12040593

**Published:** 2023-04-13

**Authors:** Marie Anne Eurie Forio, Peter L. M. Goethals, Koen Lock, Thi Hanh Tien Nguyen, Minar Naomi Damanik-Ambarita, Luis Dominguez-Granda, Olivier Thas

**Affiliations:** 1 Department of Animal Sciences and Aquatic Ecology, Ghent University, Coupure Links 653, 9000 Ghent, Belgium; peter.goethals@ugent.be (P.L.M.G.); tien.nguyenthihanh@phenikaa-uni.edu.vn (T.H.T.N.);; 2 Faculty of Biotechnology, Chemistry and Environmental Engineering, Phenikaa University, Yen Nghia, Ha Dong, Hanoi 10000, Vietnam; 3 Bioresource Research Center, Phenikaa University, Yen Nghia, Ha Dong, Hanoi 10000, Vietnam; 4 Department of Chemical and Environmental Sciences, Escuela Superior Politécnica del Litoral (ESPOL), Km 30.5, Via Perimetral, Guayaquil P.O. Box 09-01-5863, Ecuador; ldomingu@espol.edu.ec; 5 Data Science Institute, I-Biostat, Hasselt University, Agoralaan–Gebouw D, 3590 Diepenbeek, Belgium; olivier.thas@uhasselt.be; 6 National Institute for Applied Statistics Research Australia (NIASRA), University of Wollongong, Wollongong, NSW 2522, Australia; 7 Department of Applied Mathematics, Computer Science and Statistics, Ghent University, Krijgslaan 281, 9000 Ghent, Belgium

**Keywords:** flow velocity, limnology, river management, threshold values, tipping points, traits, sediments, turbidity

## Abstract

**Simple Summary:**

Questions on how features of aquatic invertebrates (e.g., size, feeding style, respiration mode) evolve along a gradient of flow velocity, turbidity and elevation are rarely addressed. Furthermore, another outstanding question is what is the environmental condition in which an abrupt change occurs in terms of the compositions and abundance of these organisms’ features. To answer the previous questions, we collected macroinvertebrates and measured the flow velocity, turbidity and elevation in the streams of the Guayas basin (Ecuador); subsequently, we analyzed the data. We observed that the abundance of most organisms’ features increased with increasing flow velocity, while they decreased with increasing turbidity. Furthermore, there was a substantial drop in the abundance of tegument-respiring invertebrates beyond the turbidity of 5 NTU and an abrupt decline in the diversity of the organisms’ features below 22 m above sea level, implying the need to focus water management in these altitudinal regions. Our results suggest that there is a need to implement measures that minimize erosion to alleviate turbidity that affects these organisms. The findings of our study provide a basis to determine critical targets for aquatic ecosystem management.

**Abstract:**

Although the trait concept is increasingly used in research, quantitative relations that can support in determining ecological tipping points and serve as a basis for environmental standards are lacking. This study determines changes in trait abundance along a gradient of flow velocity, turbidity and elevation, and develops trait–response curves, which facilitate the identification of ecological tipping points. Aquatic macroinvertebrates and abiotic conditions were determined at 88 different locations in the streams of the Guayas basin. After trait information collection, a set of trait diversity metrics were calculated. Negative binomial regression and linear regression were applied to relate the abundance of each trait and trait diversity metrics, respectively, to flow velocity, turbidity and elevation. Tipping points for each environmental variable in relation to traits were identified using the segmented regression method. The abundance of most traits increased with increasing velocity, while they decreased with increasing turbidity. The negative binomial regression models revealed that from a flow velocity higher than 0.5 m/s, a substantial increase in abundance occurs for several traits, and this is even more substantially noticed at values higher than 1 m/s. Furthermore, significant tipping points were also identified for elevation, wherein an abrupt decline in trait richness was observed below 22 m a.s.l., implying the need to focus water management in these altitudinal regions. Turbidity is potentially caused by erosion; thus, measures that can reduce or limit erosion within the basin should be implemented. Our findings suggest that measures mitigating the issues related to turbidity and flow velocity may lead to better aquatic ecosystem functioning. This quantitative information related to flow velocity might serve as a good basis to determine ecological flow requirements and illustrates the major impacts that hydropower dams can have in fast-running river systems. These quantitative relations between invertebrate traits and environmental conditions, as well as related tipping points, provide a basis to determine critical targets for aquatic ecosystem management, achieve improved ecosystem functioning and warrant trait diversity.

## 1. Introduction

Over the past several decades, aquatic organisms have been used as indicators of water quality in monitoring and assessment globally [1,2,3,4]. However, the application of these organisms as an indicator of environmental status has limitations. Biological assessment methods are commonly based on taxonomy-based metrics and do not provide a mechanistic understanding of compositional changes in lotic ecosystem functioning [5,6]. Biological traits are measurable properties of organisms, which add diagnostic and mechanistic knowledge because only organisms that possess the appropriate traits can be established in the local community [6,7,8,9]. Recently, taxonomic-free attributes (e.g., body size, abundance distribution among functional groups, functional diversity and productivity) have been applied in environmental assessment. These attributes not only reflect the taxa’s adaptation to the environment [10] but also indicate ecological resilience [11].

Management of water resources is generally more effective if they are based on a clear understanding of the mechanisms that lead to the presence or absence of species groups in the environment [12]. For instance, van der Linden et al. [13] illustrate that diversity indices based on traits provide a single value of functioning which has great applicative potential in management. Furthermore, the community tolerance concept based on traits can also assist stream managers in decision-making related to management options [14].

The application of traits in threshold-based management is underexplored. In particular, the determination of tipping points or thresholds, which is defined as a point at which a system experiences a qualitative change mostly in an abrupt and discontinuous way, facilitates the identification of regulatory limits or management thresholds [15,16]. Furthermore, the application of thresholds in management can support a more precise prioritization of management interventions based on possible ecological outcomes, and increased opportunities to improve and reach clear management targets [17]. Tipping points are also estimated to safeguard a particular group of organisms, thereby addressing the issues of resilience and sustainability [18]. Moreover, the inclusion of environmental tipping points could significantly change the cost-benefit assessments, which potentially alter the optimal policy [19]. Thus, the knowledge of these thresholds allows managers and policy-makers to deliver cost-effective solutions and strategies before an ecosystem collapse.

Traits in tropical countries are understudied and therefore little is known about the responses of macroinvertebrate traits towards flow velocity, turbidity, and accumulated impacts of human activities in these regions [20]. These environmental variables are particularly important, as studies have indicated that flow velocity, sedimentation, and multiple stressors strongly affect macroinvertebrate traits [21,22,23,24]. Although a few studies investigated the effect of flow velocity and sedimentation on macroinvertebrate traits, most are implemented in temperate regions or mesocosms [21,25,26,27]. Their studies indicated that sedimentation and reduced flow velocity strongly affect invertebrate abundance and diversity, thereby reducing functional redundancy [21]. Specifically, their findings also suggested that certain traits (i.e., sprawlers and burrowers) preferred low flows while swimmers and climbers tend to positively respond to flow velocity [25,27].

We presented a case study in a large multifunctional tropical river basin (i.e., Guayas River basin, Ecuador), aimed to investigate the changes of trait abundance along a gradient of flow velocity, turbidity, and elevation, and estimated the tipping points for each trait and trait diversity metric with respect to each of these abiotic factors. These abiotic factors are particularly important stressors within the case study basin, as various human activities such as urban-industrial development, hydroelectric power generation, agriculture, and aquaculture threaten the quality of the water and have resulted in biodiversity loss and ecological quality degradation [28,29]. The lower elevation regions of the basin suffer from the cumulative pressures of human activities, while higher regions receive lesser anthropogenic pressures. Taking this into account, elevation can be considered as a proxy for multiple stressors. We developed trait–response curves, which are a plot of the estimated abundance of a trait as a function of an environmental variable. Subsequently, tipping points for each trait and trait diversity index were estimated. We hypothesized that only certain traits respond to gradients of flow velocity, turbidity, and elevation and responses can be described in Table 1. The trait–response curves provide essential knowledge on the links between traits and environmental conditions, which not only offers insights into how the functioning of ecosystems varies at the opposite ends of major environmental gradients [30] but also contributes a better understanding of the complex relations between the abiotic and biological processes in river systems. Furthermore, the findings of this study provide insights into the potential application of macroinvertebrate traits in environmental management.

## 2. Methods

### 2.1. Study Area

**Figure 1:**
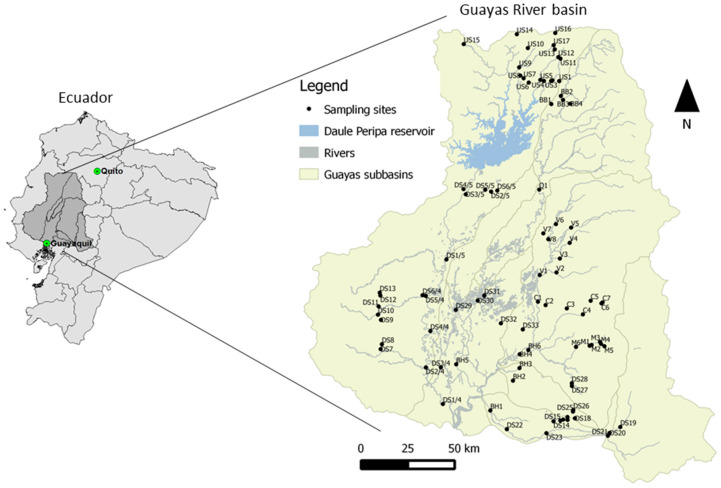
The sampling sites in the Guayas River Basin. Only the main rivers and the Daule Peripa reservoir within the basin are shown.

The Guayas River basin is located in central-western Ecuador. It is one of the major catchments in Ecuador and occupies a land surface area of 34,000 km^2^ (Figure 1). The Daule Peripa reservoir is located in the Guayas River basin and covers an area of approximately 300 km^2^ [44]. The Guayas River discharges into the Pacific Ocean. The elevation of the basin ranges from 0 to 6250 m above sea level (a.s.l.), wherein 46% of the basin has an elevation of less than 200 m a.s.l. [45]. The dry season falls between May and November.

### 2.2. Data Collection

We sampled macroinvertebrates at 88 different locations in the running waters of the Guayas River basin from October to November 2013. The sampling sites were selected to obtain a gradient of environmental variables, i.e., sites with low, moderate and high anthropogenic impacts were chosen. The sampling campaign was conducted during the dry season for safety reasons and to ensure accessibility of all sampling locations. Macroinvertebrates were collected through kick sampling with a standard handnet (conical net with a frame size of 20 × 30 cm and a mesh size of 500 µm, attached to a stick) as described by Gabriels et al. [46]. For each sampling site, a 10–20 m stretch was sampled for 5 min. Sampling effort was proportionally distributed across all aquatic habitats present at the sampling site, including bed substrate (stones, sand or mud), macrophytes (floating, submerged, emerging) and other floating or submerged natural and artificial substrates. All the collected materials were transferred to buckets with covers. Afterwards, samples were sieved (mesh size of 500 µm) and organisms were sorted alive in the laboratory and then preserved in 10 mL tubes with 96% ethanol to reach a final concentration of 70%. Macroinvertebrates were identified to the family level using the identification keys of De Pauw et al. [47] and Dominguez and Fernández [48]. After identification, the total number of individuals for each family was counted.

At each site, altitude and coordinates of latitude and longitude (based on the Geographical Coordinate system (GCS) EPSG: 4326) were recorded using Garmin-Global Positioning System (GPS) (Garmin Legend; Garmin Ltd., Olathe, KS, USA). Flow velocity at each site was measured several times at different points using a handheld Höntzsch probe (HFA-model; Höntzsch, Waiblingen, Germany). The final flow velocity value was determined from the average of the measurements taken. Before the collection of macroinvertebrates, water was collected in a pre-rinsed bucket to measure the turbidity of the water, as direct measurement in the water was not possible for some shallow stream sites. The water in the bucket was homogenized by mixing, and then the turbidity of the water was measured using the chemical probe model YSI 6600 V2 (YSI manufacturer, Yellow Springs, OH, USA). The summary statistics of the environmental variables are presented in Table 2.

### 2.3. Data Processing

#### 2.3.1. Trait Allocation

Information on traits of each macroinvertebrate was gathered from various databases (Table 3 and Table S2). As each taxon was identified to the family level, which is of a higher level than the one given in most trait databases, the traits of the most dominant and/or common genus were used, which were based on the taxonomy expert’s knowledge (K. Lock, personal communication, May 2016; Table S2). The traits of 71 percent of the taxa were found in the database of Tachet et al. [49]). In particular, the database used the fuzzy coding procedure to describe the link between a family and its traits, which provides information on the degree of taxon’s preference towards a certain trait. A score for each taxon was assigned which describes its affinity to each trait in the respective grouping feature, from ‘0’ indicating ‘no affinity’ to ‘5’ indicating ‘high affinity’ [5]. The grouping features feeding style, respiration, locomotion, reproduction and maximal size were selected as we expect that some traits within these grouping features respond to gradients of turbidity, velocity and elevation (*cf*. Table 1). Furthermore, we included as many traits as possible based on the available trait information for all the recorded taxa to provide an overall functional diversity estimate (*cf.* Section 2.3.2). A detailed description of each trait is presented in Table S1.

To provide an estimate of trait abundance, we applied a weighted method to translate the taxa’s abundance into trait abundance. We assigned each of these traits by a coefficient ranging from 0 to 1, depending on the affinity of the trait if a taxon was characterized by more than one trait within the grouping feature. For instance, the feeding strategy of a certain taxon has an affinity of 1 and 3 for deposit feeder and scraper, respectively. Therefore, a coefficient of 0.25 and 0.75 was assigned to deposit feeder and scraper, respectively. Subsequently, the abundance of each trait was obtained by multiplying the abundance (count) of the taxon and the respective coefficient. Then, the numeric abundance estimation of each trait was rounded to the nearest whole number. An example is Baetidae in site US17 has an abundance of 8 and is fuzzy coded 1 for deposit feeder and 3 for scraper. Thus, deposit feeder and scraper were assigned an abundance of 2 and 6, respectively. In each site, the total abundance of each allocated trait was summed for all taxa present. For a few taxa with traits not fuzzy coded, an equal coding was assigned to different traits. For instance, a coefficient of 0.5 was used if there were two traits involved. For further explanation of the trait abundance allocation method, we refer to Forio et al. [50].

#### 2.3.2. Calculation of Functional Diversity Indices

For each site, we calculated a set of diversity metrics, i.e., Shannon–Weaver (H; Equation (1)), Simpson (D_1_; Equation (2)), inverse Simpson (D_2_; Equation (3)), Richness (S), Pielou’s evenness (J; Equation (4)) using the vegan R package [51].

(1) $$H=-\sum_{i=1}^{S}p_{i}\;\ln\;p_{i}$$

(2) $$D_{1}=1-\sum_{i=1}^{S}p_{i}^{2}$$

(3) $$\;\;D_{2}=\frac{1}{\sum_{i=1}^{S}p_{i}^{2}}$$

(4) $$J=H/\log\;\left(S\right)\;$$

where p_i_ is the proportion of trait i, and S is the total number of traits.

### 2.4. Data Analysis

Prior to the analyses, eight cases with missing observations were eliminated resulting in 80 instances left for analysis.

#### 2.4.1. Response Curves

To relate the abundance of each trait (response variable) as a function of each environmental variable (predictor variable), negative binomial regression models (NBM) were fitted. The model assumes that for a given environmental variable, the trait abundance can be described by a negative binomial distribution. Similar to Poisson regression, the conditional mean of the negative binomial distribution is related to the environmental variables through a log-link function; the logarithm of the mean abundance can be modeled as a linear or quadratic function of one or more environmental variables. Whereas the Poisson distribution implies that the variance of the abundance equals the mean, say µ, the negative binomial distribution allows for overdispersion, i.e., the conditional variance equals µ + µ^2^/k, where k is an overdispersion parameter [52,53]. The trait absorber, asexual, hydrostatic vesicle, and size >8 cm were not included in these analyses, as most sites had zero abundance and may not follow a negative binomial distribution with respect to the environmental variables.

To relate each diversity metric (response variable) as a function of each environmental variable (predictor variable), linear regression models (LRM) were fitted. The model assumes that for a given environmental variable (X), the diversity metric (Y) is normally distributed with mean equal to the conditional mean E(Y|X) which was specified as a linear or quadratic function of the response variable.

For the trait abundance–response curves, all the model fits were performed after centering the flow velocity, turbidity and elevation (i.e., subtracting the mean), because this procedure reduces the strength of the collinearity between the terms in the statistical model, thus reducing the variance inflation effect. Backward elimination was applied as a model selection procedure to determine whether a linear or quadratic relation exists between abundance of a trait and the environmental variable, i.e., first the model with a linear and quadratic effect was fitted; if the quadratic effect was not significant at 5% level of significance, the term was removed from the model, and the linear term was tested, again at 5% level of significance. Model assumptions were assessed by plotting the deviance residuals against fitted values to assess the homogeneity and correctness of the model.

All parameters in the NBM and LRM were estimated by means of maximum likelihood [54]. All statistical tests were performed at 5% level of significance. All analyses were implemented in the R software [55] and negative binomial regression was performed with the MASS R-package [56]. To visualize the model, we plotted the results of the NBM per trait as the estimated mean abundance of the selected trait as a function of each environmental variable.

#### 2.4.2. Determination of Tipping Points

We estimated the tipping points for each trait and diversity metric with respect to flow velocity, turbidity and elevation using the segmented regression method fitted with NBM and LRM, respectively. This method is a regression model in which the relationships between the response and explanatory variables are piecewise linear, i.e., they are represented by two or more straight lines that are connected by unknown values, which are usually referred to as breakpoints or tipping points. The broken linear predictor may be defined by two-equation models before (Equation (5)) and after (Equation (6)) the tipping point (ψ) [57]. The segmented method needs a starting value to estimate tipping points. As ψ can be situated along the range of the predictor variable, we tested iteratively each potential tipping point. If the tipping point does not exist, the difference-in-slopes parameter has to be zero [58]. We applied the Davies test [59] and score test [60] to check for a non-zero difference-in-slope parameter of a segmented relationship for an LRM and NBM model fit, respectively, at 5% level of significance. Davies and score test provided “best” tipping point values; however, we selected the final tipping point based on the estimation by the segmented method, which is closest to the best value provided by the Davies or score test [58,60]. These analyses were performed in the R software [55] using the Segmented R package and the functions segmented, summary.segmented, print.segmented, pscore.test and davies.test of the package [58].

If x < ψ, then y = *α + β*_1_x (5)

If x > ψ, then y = (*α − β*_2_ ψ) + (*β*_1_ + *β*_2_)x (6)

where *α* is the left side intercept, *β*_1_ is the slope parameter of the linear regression at the left side of tipping point, *β*_2_ the difference in slope parameter between the two linear regressions around the tipping point, ψ is the tipping point, x is the predictor variable and y is the response variable.

## 3. Results

### 3.1. Overview of Ecuadorian Traits Data

Many taxa were predators (44%), crawlers (70%), reproduce in clutches (cemented; 47%), respire by gills (57%) and tegument (63%), and invertebrates with sizes of >0.5–1 cm (40%) and >1–2 cm (40%) (Figure S1). Accordingly, the most abundant trait over all the sampled locations were also crawlers, clutches (cemented)-reproducing invertebrates, gill- and tegument-respiring invertebrates, and invertebrates with size >1–2 cm (Figure S2). Scrapers and taxa that were temporarily attached were also abundant.

### 3.2. Trait Abundance Models

**Figure 2:**
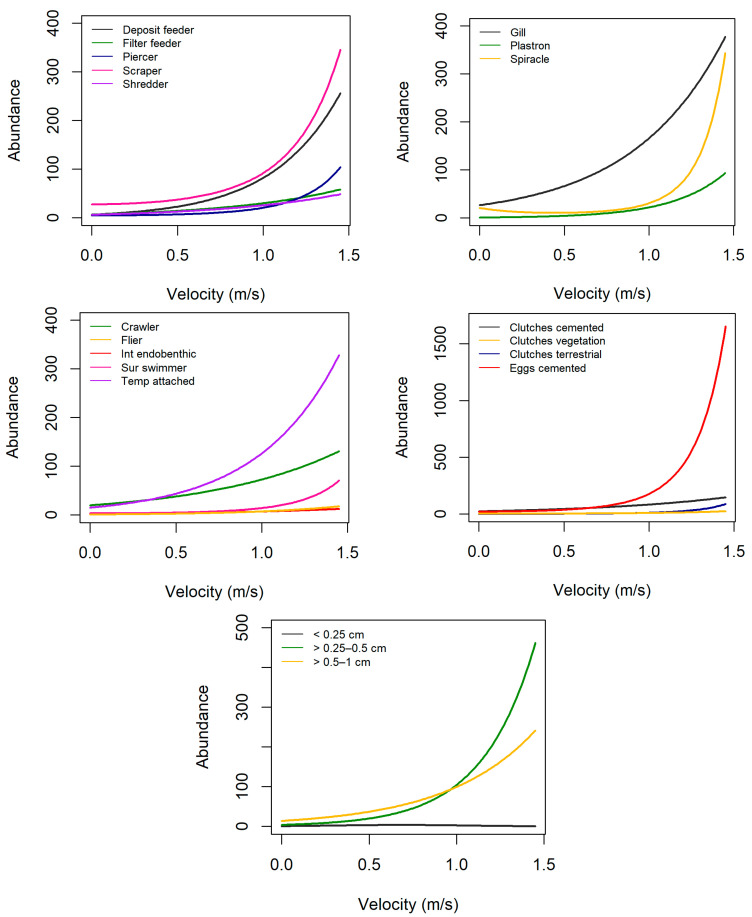
Estimated mean abundance of each trait in relation to velocity.

**Figure 3:**
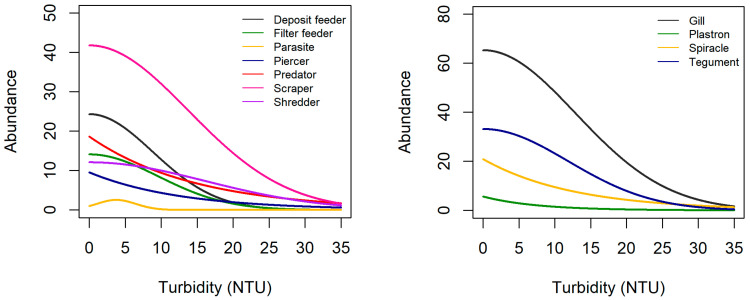

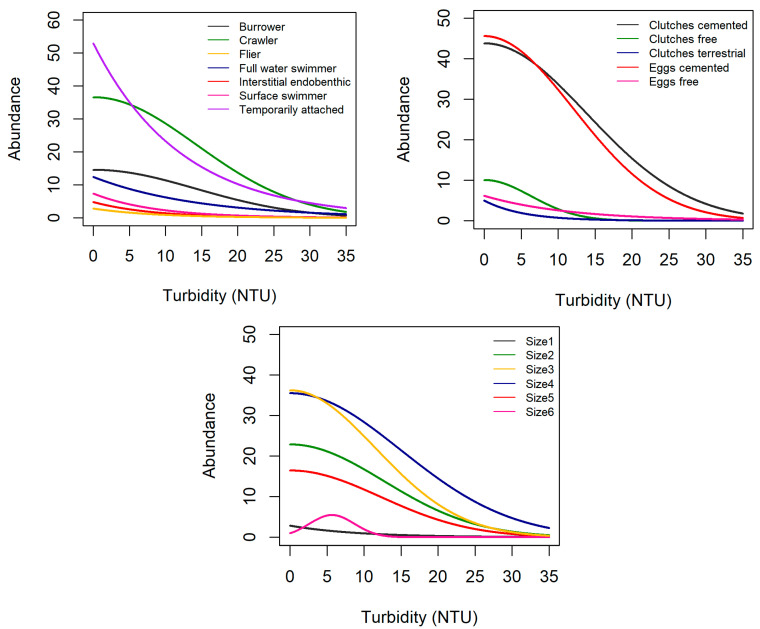
Estimated mean abundance of each trait in relation to turbidity.

**Figure 4:**
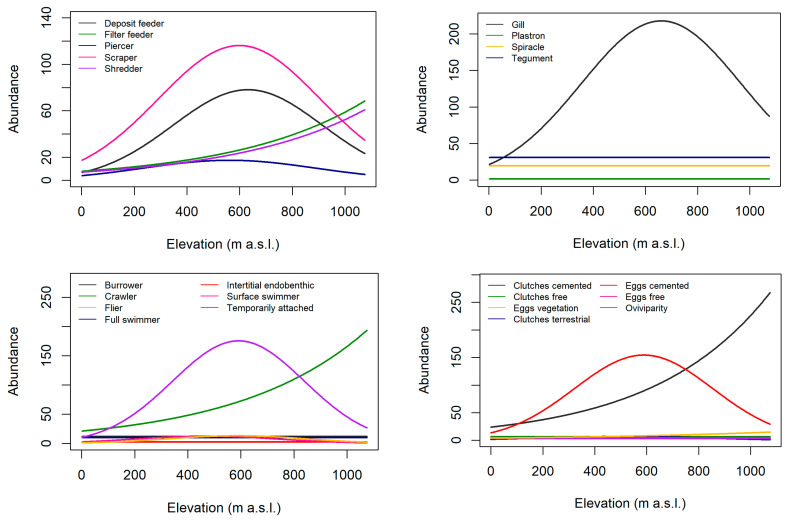

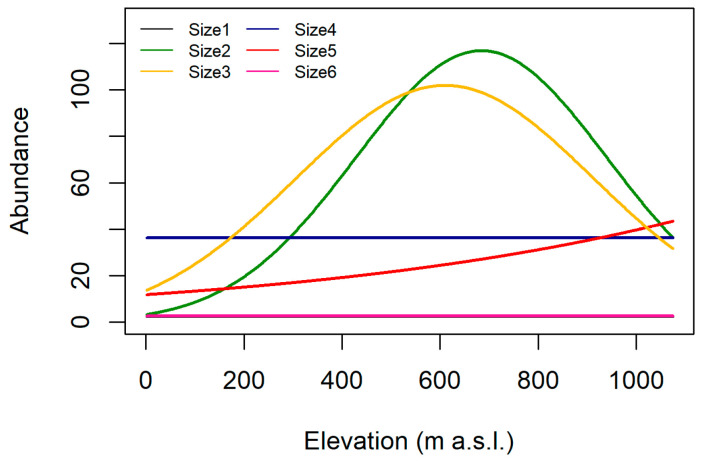
Estimated mean abundance of each trait in relation to elevation (m above sea level) where size1, size2, size3, size4, size5 and size6 refers to size ≤0.25 cm, >0.25–0.5 cm, >0.5–1 cm, >1–2 cm, >2–4 cm and >4–8 cm, respectively. The mean abundance of size1 was mostly overlaid by size6.

In general, the abundance of most traits increased with increasing velocity (Figure 2, Tables S3–S7). A substantial increase in abundance occurs for several traits at a flow velocity higher than 0.5 m/s, and this is even more substantially noticed at values higher than 1 m/s. Scrapers, taxa respiring by gills, taxa that were temporarily attached and taxa reproducing through cemented isolated eggs had the highest mean abundance. Invertebrates with sizes >0.5–1 cm had the highest mean abundance at velocities 0 to 0.9 m/s, while invertebrates with sizes >0.25–0.5 cm had the highest mean abundance at velocities 0.9 to 1.5 m/s. Responses along the gradient of flow velocity were relatively constant for invertebrates that are fliers, interstitial endobenthic, small-sized invertebrates (<0.25 cm), as well as those that reproduce through clutches in vegetation. Crawlers and invertebrates reproducing through cemented clutches and terrestrial clutches slightly increase in abundance at high flow velocities. In contrast, the abundance of most traits decreased with increasing turbidity (Figure 3). In general, the mean abundance of most traits generally decreases abruptly between 5 and 10 NTU. The mean abundance of deposit feeders, scrapers, piercers, taxa respiring by gills, taxa that are temporarily attached, taxa reproducing through isolated eggs (cemented) and invertebrates with sizes >0.5–1 cm and >0.25–0.5 cm peaked at about 450 to 700 m above sea level (a.s.l.) (Figure 4). On the other hand, the mean abundance of filter feeders, shredders, crawlers, taxa reproducing through clutches (cemented) and invertebrates with size >4–8 cm increased with increasing elevation. Mean abundance of tegument-, spiracle- and plastron-respiring invertebrates, invertebrates with sizes ≤ 0.25 cm, >1–2 cm and >4–8 cm were constant across the altitude range. Mean abundance along a gradient of elevation is also relatively constant for most reproduction traits except cemented eggs and clutches and locomotion traits except crawlers and temporarily attached invertebrates.

### 3.3. Tipping Points

**Figure 5:**
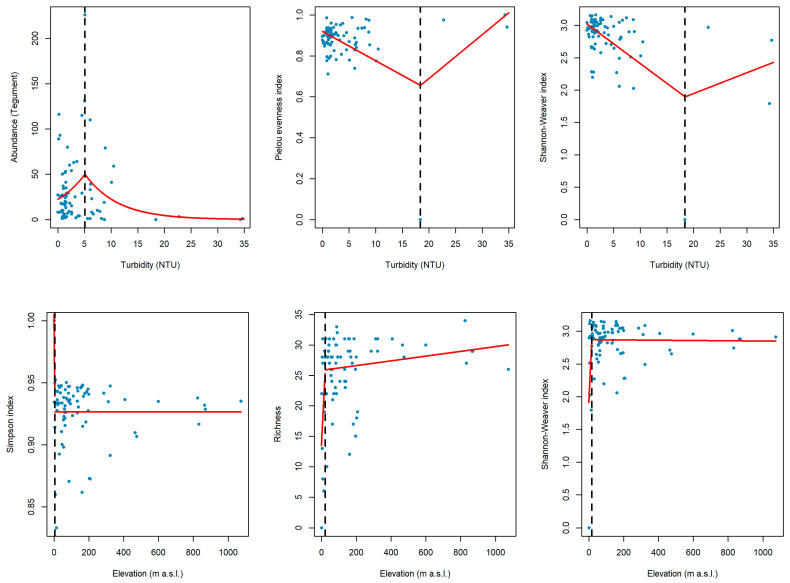
Estimated tipping points (broken black line) for tegument’s abundance, Pielou’s evenness index and Shannon–Weaver index in relation to turbidity and for Simpson index, trait richness and Shannon–Weaver index in relation to elevation. The blue dots represent the data while the red lines represent the linear model estimated by the segmented regression method.

Statistically significant tipping points (ψ) were found for tegument’s abundance, Pielou’s evenness index and Shannon–Weaver index with respect to turbidity and for Simpson index, trait richness and Shannon–Weaver index with respect to elevation (Figure 5 and Table S8). It is observed that the abundance of tegument increases as the turbidity increases until 5 NTU and decreases after 5 NTU. Both the Pielou’s evenness and Shannon–Weaver values decrease as turbidity increases until 18.4 NTU and increase after 18.4 NTU. However, the right-side linear models only consist of a few data points (i.e., 3) and can be deemed less meaningful. A sharp decrease was observed for Simpson index from 0 to 6 m a.s.l, then it remained constant after 6 m a.s.l. The trait richness was increasing as the elevation increased but a sharp decrease was observed below 22 m a.s.l. Lastly, Shannon–Weaver index sharply decreases below 18 m a.s.l. but is relatively constant above 18 m a.s.l.

## 4. Discussion

### 4.1. Ecological Insights

Organisms are filtered to their habitats through their traits, that is, only the organisms that possess suitable traits can pass these filters and establish themselves in the local community [6]. As exemplified in our study, traits shifted along a gradient of flow velocity, turbidity, and elevation.

Reduced flow velocity can result from decreases in water flow, thereby diminishing water depth and wetted channel width, increasing sedimentation, and changing the thermal regime and water chemistry [61]. Reduced flow velocity also affects the diversity, composition and abundance of aquatic invertebrates [21,32,62]. Impacted rivers with low flow velocities tend to have poor ecological status [28,63]. The results of our study indicate that as flow velocity increases, the abundance of most traits increases. Most of the expected responses are in line with our findings. The abundance of filter feeders is positively associated with flow velocity as higher flow velocities ensure adequate nutritional requirements for these organisms [32,33]. Higher flow velocities are also favorable for the growth of periphyton and benthic biofilms [34], which guarantees adequate food for scrapers. It was expected that the abundance of deposit feeders and shredders decline with increasing flow velocity due to resuspension and drifting of their food sources, respectively; however, the opposite occurred in our study. A likely explanation for this phenomena is the presence of microhabitats in the study sites such as pools, where their food sources are deposited or accumulated, thereby providing them with their nutritional needs. More gill-respiring invertebrates were observed at higher flow velocities and are in line with our expected outcome. Although no notable response was observed for tegument-respiring invertebrates along the gradient of flow velocity, this outcome is in line with the findings of Brooks et al. [64]. The a priori predicted finding is that fewer tegument-respiring invertebrates will be observed at low flow velocity [35], but as suggested by our own and Brooks et al.’s [64] findings, this expected response may be simplistic, and tegument-respiring invertebrates’ response to flow velocity is likely more complex than perceived, which could potentially be due to other behavioral (e.g., body undulation) and physiological (e.g., respiratory pigments) strategies providing sufficient respiratory capacity during low flow velocities [65]. As predicted, small-sized invertebrates increase in abundance as flow velocity increases, given that smaller body size has lower drag force at high flow velocities [36]. Likewise, as expected, invertebrates reproducing by cemented eggs and clutches tend to increase in abundance as flow velocity increases because they secure their eggs or clutches against the high flow velocity by cementing them [37]. On the other hand, no significant responses were observed for the abundance of free eggs and clutches along the gradient of flow velocity. We speculate that some invertebrates situate their eggs or clutches in pool microhabitats to protect them from drifting. Temporarily attached invertebrates increase in abundance as the flow velocity increases, potentially due to their resistance to drifting. Our findings on responses of crawlers with flow velocity are in line with the observations in Horrigan and Baird [38], in which more crawlers are found at high flow velocities. Surprisingly, the abundance of surface swimmers was positively correlated to flow velocity, which is contradictory to the expected outcomes. We speculate that the presence of pool microhabitats and the abundance of food resources provide favorable conditions for these animals to flourish even at high flow velocities. In general, many traits are highly abundant at high flow velocities, suggesting that at sites with high flow velocities, there are more available functions and are, therefore, potentially more resilient to stressors, as sites with functional redundant taxa (i.e. taxa performing similar functions) have the capacity of buffering the functions of lost taxa, thereby promoting stability and resilience to disturbance [66].

Turbidity has been known to be negatively associated with macroinvertebrate communities [28,67,68]. Possible causes of turbidity in streams are soil erosion, waste discharge, urban runoff, silt and clay, eroding stream banks, and excessive algal growth [69,70]. In our study, we observed that the abundance of most traits decreased with increasing turbidity. Fine sediment deposition—one of the potential causes of turbidity, generally results in a decrease in total invertebrate abundance [21]. Specifically, fine sediments (defined as inorganic and organic particles <2 mm diameter) cause abrasion to invertebrates, physical damage to the invertebrates’ breathing apparatus and potentially clog their body parts, such as gills and filter-feeding apparatus [71]. The transport of fine particles can result in a build-up on the organs, which disrupts the normal functioning of the organisms. Fine sediments also impair habitats by clogging the interstices and reducing oxygen levels in the hyporheic zone, decrease food availability by attenuating light needed for optimal algal growth and covering algal food for grazers, and reduce the quality and palatability as well as access to food for shredders [72,73,74,75]. Sedentary invertebrates and cemented eggs or clutches are also affected by fine sediment deposition by being buried, leading to a lack of access to food and a reduced oxygen supply [71]. Our findings, however, suggest that not only sedentary invertebrates are affected by turbidity but also mobile taxa and different invertebrate sizes. Some of these traits are less likely to be affected by turbidity but the organisms possessing these traits may also possess other traits that are sensitive to turbidity (fine particles), thereby affecting the general abundance of invertebrates. Contrary to the predicted response, we observe that the abundance of deposit feeders is negatively associated with turbidity and, in particular, abruptly declines at about 5–10 NTU. We speculate that the high load of fine particles in turbid waters is causing more harm to the deposit feeders offsetting the benefits of food provisioning to these organisms. Similar to streams with high flow velocities, waters with low turbidity (<10 NTU) potentially promote stability and resilience to disturbances.

A shift of traits is observed along an altitudinal gradient. Elevation has been reported as the key environmental factor affecting aquatic invertebrates [28,76,77,78]. It is observed that rivers at the upstream higher elevation have a different community composition and are moreover less impacted compared to downstream systems [28], and likely the functioning is similarly affected. Likewise, Rezende et al. [40] and Feio et al. [41] found increased taxonomic richness and density of macroinvertebrate communities at higher altitudes. Consequently, these shifts in taxonomic diversity and composition in response to elevation are often confounded by the impacts of human activities, which are often most extensive at the lower altitudes [79,80,81]. Our findings suggest that the decrease in abundance of several traits (e.g., crawler, cemented eggs) at lower elevations is a result of the cumulative impacts of intensive anthropogenic activities and to a lesser extent the natural river characteristics at these locations. It is worth noting that our findings tend to follow the predictions of the River Continuum Concept (RCC, Vannote et al. [31]): shredders increased in abundance as the altitude increased because more leaf litter input is present, which is their food, and scrapers and deposit feeders peaked at mid-elevation as more of their food is available in the midstreams. Contrary to the prediction of the RCC, the reduction in deposit feeder abundance is prominent at low elevation. This is likely due to more intense human activities and impacts at low elevations, making it less suitable for most invertebrates to live. Similar to the findings of Statzner et al. [42], we also observed that crawlers tend to increase at increasing altitudes, which is due to coarser and stable substrates in the higher altitudes allowing for these organisms to effectively grip [82]. More invertebrates that reproduce through cemented clutches are also observed at high elevations. This can be explained by the presence of harsher floods in small streams at high altitudes, which are generally viewed as a disturbance [42]. Invertebrates address these disturbances through resistance and resilience by better attachment to the stream bottom [43].

### 4.2. Application in Environmental Management

Our study indicates the associated effect of flow velocity and turbidity on traits: low flow velocity and turbid waters support fewer traits. Resolving issues on turbidity and flow velocity may lead to better aquatic ecosystem functioning and overall ecological resilience. Potential causes for low flow velocity are water regulations such as hydropower dam implementation (location and design) and operation, as well as water abstraction. Thus, it is important to establish minimal flow requirements within the basin. Turbidity within the basin is potentially caused by erosion, including bank erosion, due to agricultural activities. This implies that measures that can reduce or limit erosion should be implemented in the agricultural areas within the basin. Among these measures are cover crops, conservation crop rotation, buffer strips, contour farming, riparian forest buffers and sediment basins [83,84]. These measures must be prudently selected and strategically implemented to optimize their cost-effectiveness.

### 4.3. Tipping Points in Water Management

Detecting thresholds is highly relevant to promote environmental protection; it is noteworthy that the management of ecosystems is most effective when crossing the thresholds is avoided [17]. Our study estimated the tipping points when the function begins to disappear or abruptly change using the segmented regression method. This method provides simple and easy-to-interpret outcomes, which are facilitated by the plotted graphs. The method also provides a standard error that shows a range where the tipping point is situated and it is also flexible with the probability distribution of the data (e.g., be it Gaussian or Negative binomial or Poisson or other distribution) with respect to the predictor variable. Other regression-based methods exist, such as Fstat [85] and threshold by mean (TMean; Robin et al. [86]); however, Vanacker et al. [57] indicated that, among the approaches, the segmented regression method is the best method for evaluating tipping points, as the approach provides a more biological meaningful tipping point. Another approach for estimating the community threshold is Threshold Indicator Taxa ANalysis (TITAN), which detects changes in taxa distributions along an environmental gradient and evaluates synchrony among taxa change points as an indication for community thresholds [87]. The threshold value estimated by the three regression-based methods is not comparable to TITAN as the latter detects synchronous taxa change at a particular level of an environmental gradient as an indication for community thresholds. One of the limitations of TITAN is it is only intended for (taxa) abundance data and not for community metrics (e.g., diversity indices, number of Ephemeroptera, Plecoptera, and Trichoptera taxa (EPT)) because TITAN was designed based on a negative binomial distribution of response variables [88] while these community metrics usually follow other probability distributions with respect to an environmental variable.

In our study, the significant tipping point represents a threshold along a gradient of an environmental variable (i.e., turbidity or elevation) where a large change in diversity metric or trait abundance is observed. We identified a significant tipping point for turbidity in relation to the abundance of tegument (i.e., 5 NTU) which is relatively low. It is, however, observed that most instream waters within the basin had turbidity lower than 10 NTU, suggesting that most of these waters were relatively clear and only downstream sites were in general turbid. As tegument-respiring invertebrates are expected to rapidly decrease with an increasing action of a stressor ‘oxygen deficit’ [35], we speculate that the particles causing turbidity (e.g., clay or fine sediments) damage the breathing apparatus of the tegument-respiring invertebrates, which caused their decline in abundance after 5 NTU.

Significant tipping points were also identified for elevation, wherein an abrupt decline in trait richness and Shannon–Weaver index was observed below 22 m a.s.l. and 17.5 m a.s.l., respectively. The altitude of the basin ranges from 0 to 6250 m a.s.l.; however, 46% of the basin has an altitude of less than 200 m a.s.l. and the sampling sites were in the range of 2 to 1075 m a.s.l. Within the basin, human activities generally upsurge as the elevation decreases and so does the impacts of these activities, which accumulate at lower altitudes and are the likely explanation for the rapid decline of the trait diversity indices at lower elevation. This implies that management measures are needed particularly at lower altitudes below 22 m a.s.l. to reduce the impacts of human activities on the aquatic ecosystems.

In our study, the identification of tipping points or thresholds provides potential targets for aquatic ecosystem management facilitating more informed decisions to achieve improved ecosystem functioning. Furthermore, thresholds are required in commonly developed decision support tools to define a high or low value for an environmental variable of concern [89]. Thresholds are also essential for water managers due to the possible high risk of negative consequences when these thresholds are continuously exceeded, which may limit future management actions and may potentially be non-reversible [90].

### 4.4. Implications for Further Studies

Although the data were collected only at one sampling event, it provided significant ecological insights into the responses of traits to gradients of flow velocity, turbidity and elevation. As the temporal variation of trait–environment relationships is understudied [91], we recommend collecting data at different periods (i.e., yearly and seasonal).

Another limitation of our study is sites had variable stream orders and widths, which range from 2 to 40 m and a few sites with widths between 60 and 100 m. This variability between sites may have affected trait composition and abundance as studies have suggested that stream order and width explain the distribution of macroinvertebrates [92,93]. Furthermore, a study indicated that macroinvertebrate trait composition in the Finnish boreal headwater streams is attributable to stream width [30]. While stream width is most likely affecting the feeding styles, as suggested by the River Continuum Concept [31], related studies are scant on whether stream width and order are affecting the composition, abundance and diversity of other traits, particularly in tropical streams. Thus, future studies investigating these aspects in various regions would be beneficial for a better understanding of ecosystem functioning in lotic systems.

In our study, macroinvertebrate traits were determined based on family-level identification. This procedure was due to the limited availability of taxonomic keys in the tropics, the sparsity of trait databases for tropical taxa, and the lack of finer-level taxonomic identification of taxa and their distribution within the case study region. The availability of this information could have facilitated the assignment of more accurate affinity scores as implemented in the study of Twardochleb et al. [94]. Although family-level taxonomic data are perceived to retain much of the accuracy in functional structure and are sufficient in trait-based investigations [23,95,96], studies indicate that finer taxonomic resolution allows more discriminatory power for various community analyses [25,39,97,98]. We acknowledge that the use of family-level taxonomic data will likely lead to a slight loss of information [23]; nevertheless, our results provide sensible insights into the responses of traits to gradients of flow velocity, turbidity and elevation. Moreover, our study does not take into account the switching of traits by some organisms as a result of certain environmental conditions. For instance, taxa might change their feeding style from filtering to grazing when suspended sediment loads are high, possibly due to physical damage from particles [71]. These observations, however, are best determined through experimental set-ups. Future studies, depending on the research goals, can opt to directly measure or determine some traits, such as body size and length, to eliminate the need for taxonomic identification.

The application of traits in environmental management needs further investigation, particularly in relation to ecosystem functioning and services in the context of sustainable development [99,100]. For instance, characterizing functional composition and diversity at the natural or semi-natural environmental conditions would aid in setting target conditions for environmental management. Specifically, the functional composition/diversity at the reference condition is compared with potentially disturbed sites. However, this remains a challenge, as locations with semi-natural conditions are hard to find and access due to the increasing exploitation of natural resources even at upstream river stretches. Furthermore, there is no consensus on which traits have to be included in estimating trait or functional diversity [101]. Perhaps an option would be the development or application of stress-specific indicators based on traits such as SPEAR_pesticides_ [102]. Nevertheless, this may be limited by the possible correlation of stress-specific indicators with other indicators [103]. Thus, there is a need to test whether a developed stress-specific indicator is correlated with other indicators. On the other hand, the findings of this study can serve as reference information and the methodology can be extended to other traits (e.g., dissemination mode, life cycle durations) to further identify trait–environment relationships, which can assist in the development of stress-specific indicators or trait indicators detecting environmental degradation. Studies also indicate that traits will depend on local system composition and can drastically be affected by connectivity aspects, particularly in the case of highly isolated islands. Consequently, island river systems may have very low resilience, both in biodiversity and functioning, and in that aspect, trait-based approaches might be valuable early warning systems [104,105].

## 5. Conclusions

Our findings indicate that low flow velocity and turbid waters support fewer traits. We also identified a significant specific tipping point for turbidity in relation to the abundance of tegument, in which the abundance of tegument suddenly declines after 5 NTU. These findings suggest that measures mitigating the issues related to turbidity and flow velocity may lead to better aquatic ecosystem functioning and promote stability and resilience to disturbance. Significant tipping points were also identified for elevation, wherein an abrupt decline in trait richness was observed below 22 m a.s.l., implying that water management and mitigation measures need to be prioritized in areas with these altitudinal ranges. The identification of tipping points provides a good basis for setting potential targets for aquatic ecosystem management facilitating more informed decisions to achieve improved ecosystem functioning.

## Figures and Tables

**Table 1:** Expected trait responses to flow velocity, turbidity and elevation with increasing (+), decreasing (−), hump-shaped (h), increases until mid-elevation, then constant after mid-elevation (↑-> mid, C after mid), low abundance at low elevation (L -> low) responses and no responses (nr). The expected outcome is based on the findings of other studies and concepts [21,28,31,32,33,34,35,36,37,38,39,40,41,42,43]. The description of each trait is presented in Table S1.

Grouping Features	Traits	Velocity (+)	Turbidity (+)	Elevation (+)
Feeding style	Deposit feeder	–	+	h
	Filter feeder	+	–	h
	Parasite	nr	nr	nr
	Piercer	nr	nr	h
	Predator	–	–	↑-> mid, C after mid
	Scraper/grazer	+	–	h
	Shredder	–	–	+
Locomotion	Burrower	nr	+	–
	Crawler	+	–	+
	Flier	nr	–	↑-> mid, C after mid
	Full water swimmer	–	–	↑-> mid, C after mid
	Instertitial (endobenthic)	nr	nr	nr
	Surface swimmer	–	nr	↑-> mid, C after mid
	Temporary attached	nr	–	h
Reproduction	Clutches, cemented	+	–	+
	Clutches, free	–	–	h
	Clutches in vegetation	nr	–	L -> low
	Clutches, terrestrial	nr	nr	L -> low
	Isolated eggs, cemented	+	–	+
	Isolated eggs, free	–	–	h
	Oviviparity	nr	nr	L -> low
Respiration	Gills	+	–	+
	Hydrostatic vesicle	nr	nr	nr
	Plastron	nr	–	nr
	Spiracle	nr	nr	nr
	Tegument	+	–	+
Maximal potential size	Size1 (≤0.25 cm)	+	nr	+
	Size2 (>0.25–0.5 cm)	+	nr	+
	Size3 (>0.5–1 cm)	+	–	h
	Size4 (>1–2 cm)	+	–	h
	Size5 (>2–4 cm)	nr	–	h
	Size6 (>4–8 cm)	nr	–	h
	Size7 (>8 cm)	nr	–	–

**Table 2:** Measured environmental variables.

	Unit	Mean	Median	Min.	Max.	Standard Deviation
Elevation	m a.s.l.	166	86	2.0	1075	222
Velocity	m/s	0.3	0.3	0.0	1.5	0.29
Turbidity	NTU	4.2	2.0	0.0	34.7	6.2

**Table 3:** List of grouping features and traits considered in our study. The description of each trait is presented in Table S1.

Grouping Features	Traits
Feeding style	Absorber, deposit feeder, filter feeder, parasite, piercer, predator, scraper, shredder
Respiration mode	Gill, plastron, spiracle, tegument
Locomotion mode	Burrower, crawler, flier, full water swimmer, interstitial endobenthic, surface swimmer, temporarily attached
Reproduction mode	Clutches cemented, clutches free, clutches terrestrial, clutches in vegetation, isolated eggs cemented, isolated eggs free, oviviparity
Sizes	Size1 (≤0.25 cm), size2 (>0.25–0.5 cm), size3 (>0.5–1 cm), size4 (>1–2 cm), size5 (>2–4 cm), size6 (>4–8 cm), size7 (>8 cm)

## Data Availability

The data presented in this study are available on request from the corresponding author.

## Supplementary Materials

The following supporting information can be downloaded at: https://www.mdpi.com/article/10.3390/biology12040593/s1, Figure S1: Number of taxa linked with each trait of feeding strategy (blue), locomotion (orange), red (reproduction), respiration (violet) and size (green) with size1 (≤ 0.25 cm), size2 (>0.25–0.5 cm),size3(>0.5–1 cm), size4 (>1–2 cm), size5 (>2–4 cm), size6 (>4–8 cm) and size7 (>8 cm); Figure S2: Total abundance of each trait for feeding strategy (blue), locomotion (orange), reproduction (red), respiration (violet) and size (green) with size1 (≤ 0.25 cm), size2 (>0.25–0.5 cm), size3 (>0.5–1 cm), size4 (>1–2 cm), size5 (>2–4 cm), size6 (>4–8 cm) and size7 (>8 cm) at all sampling locations; Table S1: Traits of macroinvertebrates used in this study; Table S2: Trait allocation for each taxa in Ecuador (Ec) with trait classes of absorber (Ab), deposit feeder (DF), filter feeder (FF), parasite (Par), piercer (Pie),scraper (Scr) and shredder (Shr) for feeding strategy, burrower (Bur), crawler (Cra), flier (Fli), full water swimmer (FWS), interstitial endobenthic (IE), permanently attached (PA), surface swimmer (SS) and temporarily attached (TA) for locomotion, asexual reproduction (AS), clutches & cemented (CC), clutches & free (CF), clutches in vegetation (CV), clutches in terrestrial (CT), isolated eggs & clutches (IEC), isolated eggs & free (IEF) and oviviparity for reproduction, gills (Gil), hydrostatic vesicle (HV), plastron (pla), spiracle (Spi) and tegument (Teg) for respiration, 1 (≤0.25 cm), 2 (>0.25–0.50 cm), 3 (>0.50–1.0 cm), 4 (>1.0–2.0 cm), 5 (>2.0–4.0 cm), 6 (>4.0–8.0 cm) and 7 (>8.0 cm) for maximal potential size; Table S3: Estimates and *p*-values of each feeding strategy; Table S4: Estimates and *p*-values of each respiration mode; Table S5: Estimates and *p*-values of each locomotion mode; Table S6: Estimates and *p*-values of each reproduction mode; Table S7: Estimates and *p*-values of each size category; Table S8: Estimated tipping points with their standard errors in parenthesis of each feeding strategy trait with respect to elevation, velocity and turbidity. References [49,106,107,108,109,110,111,112,113,114,115,116,117,118] are cited in the Supplementary Materials.
