# Supplementary material for: Determining Tipping Points and Responses of Macroinvertebrate Traits to Abiotic Factors in Support of River Management
Source: Biology (Basel). 2023 Apr 13;12(4):593. doi: 10.3390/biology12040593 (PMC10135673; doi:10.3390/biology12040593)
Supplement: Supplementary file 1 [file biology-12-00593-s001.zip › biology-2244218-supplementary.pdf]

Supplementary materials

Supplementary materials for **Determining tipping points and responses of macroinvertebrate traits to abiotic factors in support of river management**

Marie Anne Eurie Forio [1,*], Peter L. M. Goethals [1], Koen Lock [1], Thi Hanh Tien Nguyen [1,2,3], Minar Naomi Damanik-Ambarita [1], Luis Dominguez-Granda [4] and Olivier Thas [5,6,7]

[1] Department of Animal Sciences and Aquatic Ecology, Ghent University, Coupure Links 653, 9000 Ghent, Belgium; peter.goethals@ugent.be (P.L.M.G.); koen_lock@hotmail.com (K.L.); tien.nguyenthihanh@phenikaa-uni.edu.vn (N.T.H.T.); minarnaomi.damanikambarita@ugent.be (M.N.D.-A.)

[2] Faculty of Biotechnology, Chemistry and Environmental Engineering, Phenikaa University, Yen Nghia, Ha Dong, 10000 Hanoi, Viet Nam

[3] Bioresource Research Center, Phenikaa University, Yen Nghia, Ha Dong, 10000 Hanoi, Viet Nam

[4] Department of Chemical and Environmental Sciences, Escuela Superior Politécnica del Litoral (ESPOL), Km 30.5, Via Perimetral, P.O. Box 09-01-5863, Guayaquil, Ecuador; ldomingu@espol.edu.ec

[5] Data Science Institute, I-Biostat, Hasselt University, Agoralaan–Gebouw D, 3590 Diepenbeek, Belgium; olivier.thas@uhasselt.be

[6] National Institute for Applied Statistics Research Australia (NIASRA), University of Wollongong, Wollongong, NSW 2522, Australia

[7] Department of Applied Mathematics, Computer Science and Statistics, Ghent University, Krijgslaan 281, 9000 Ghent, Belgium

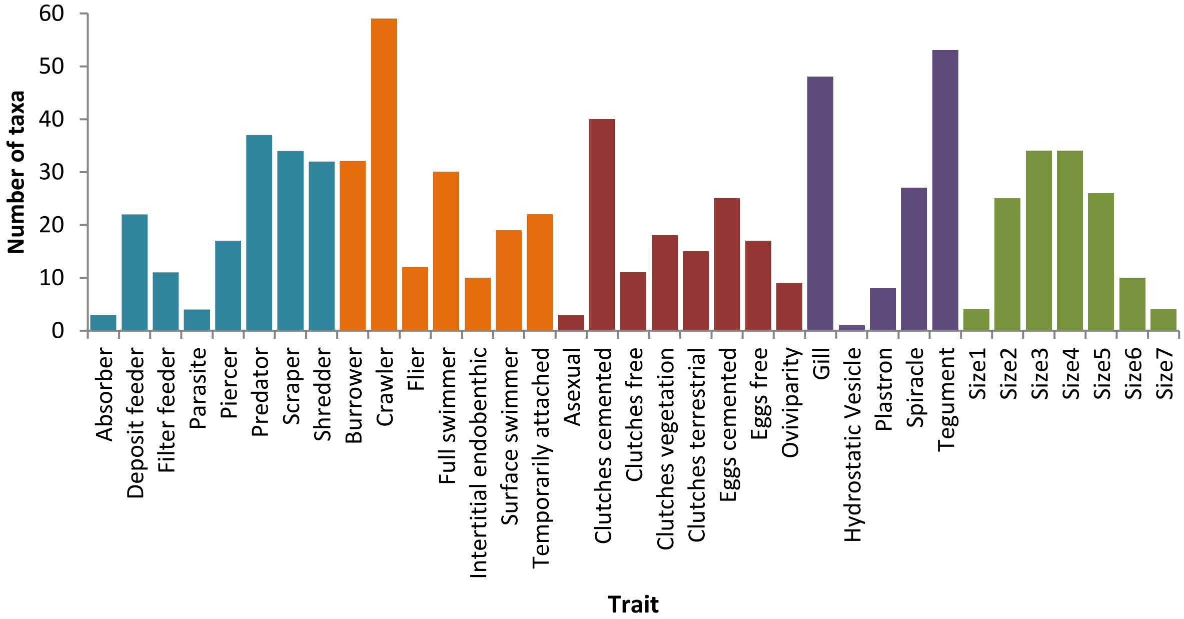

Figure S1. Number of taxa linked with each trait of feeding strategy (blue), locomotion (orange), red (reproduction), respiration (violet) and size (green) with size1 (≤ 0.25 cm), size2 (> 0.25–0.5 cm), size3(> 0.5–1 cm), size4 (> 1–2 cm), size5 (> 2–4 cm), size6 (> 4–8 cm) and size7 (> 8 cm).

Supplementary materials

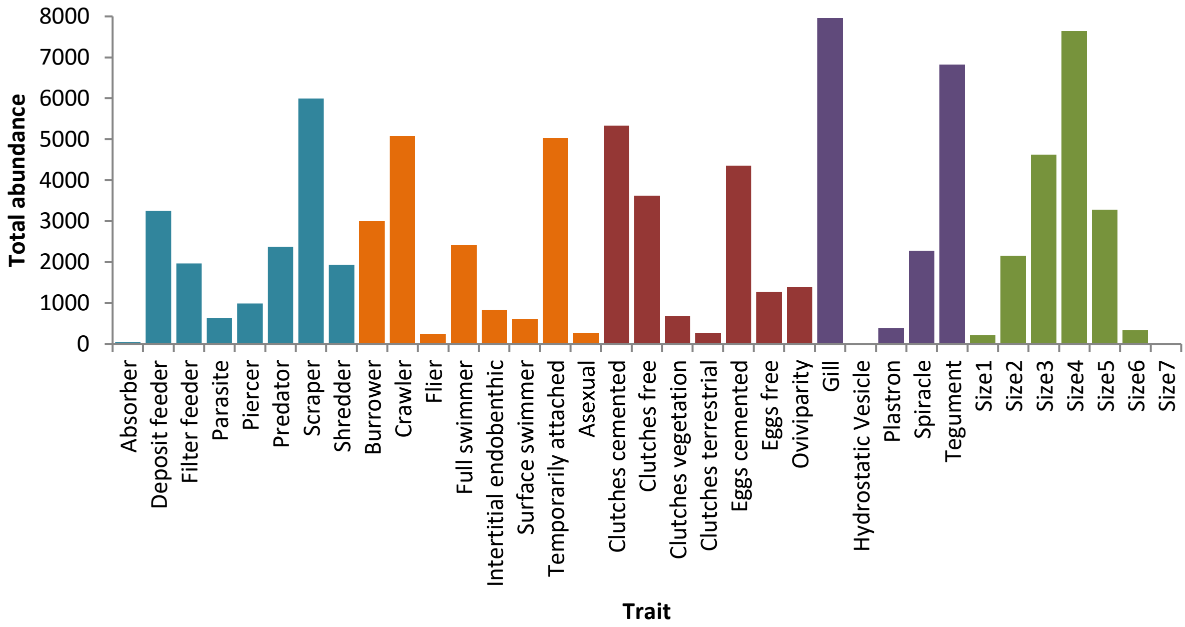

Figure S2. Total abundance of each trait for feeding strategy (blue), locomotion (orange), reproduction (red), respiration (violet) and size (green) with size1 (≤ 0.25 cm), size2 (> 0.25–0.5 cm), size3 (> 0.5–1 cm), size4 (> 1–2 cm), size5 (> 2–4 cm), size6 (> 4–8 cm) and size7 (> 8 cm) at all sampling locations.

Table S1. Traits of macroinvertebrates used in this study. Trait categories are based on the classification in Tachet, Richoux, Bournaud, and Usseglio-Polatera (2000).

Traits	Categories	Definition
Feeding type	Absorber	Absorb macromolecules through their teguments
	Deposit feeder	Feed on organic particles that are deposited on the sediment or take up sediment
	Shredder	Ingest or feed on coarse particulate organic material (CPOM) such as small sections of leaves
	Scraper/grazer	Feed on fine organic debris, consisting of microphytes and microinvertebrates, can form very fine deposits on the water surface (neuston), on hard substrates (perilithon) or on macrophytes (periphyton)
	Filter feeder	Feed on fine organic debris, microphytes and microinvertebrates present in the water column: it is called plankton in lakes and ponds and seston in running waters. Filtration happens with the aid of morphological structures or filtration through secretion of filtration structure
	Piercer	Food source is liquid or liquefied by enzymatic action. The food can be of vegetal origin, *e.g.* cellular content of filamentous algae or of animal origin
	Predator	Food source consists of an animal (micro- or macroinvertebrate) which is either swallowed whole (swallowers) or cut/ripped into pieces. Many predatory insects have specializations that are either anatomical or behavioral (i.e. plankton fishing web)
	Parasite	Lives in or on another organism (its host) and benefits by deriving nutrients from the host organism

Supplementary materials

Locomotion	Flier	Organisms that mainly have an aquatic lifestyle, but which are capable of flying by their own means. Organisms that are transported by birds.
	Surface swimmer	These are organisms that move over the water surface, organisms that swim under the surface, or organisms that hang just beneath the surface.
	Full water swimmer	These are planktonic organisms (reduced swimming capacity or nektonic, organisms with better developed swimming capacity)
	Crawler	Animals that move over the substrate
	Burrower	Animals that spend the largest part of their life burrowed into the sediment at a few centimetres from the surface
	Instertitial (endobenthic)	Epibenthic burrowers can dig themselves very deeply into the sediment
	Temporary attached	Organisms that can leave their substrate
Reproduction	Oviviparity	Embryos develop inside eggs remain in the body until the eggs are ready to hatch.
	Isolated eggs, free	Single eggs and not cemented
	Isolated eggs, cemented	Single eggs and cemented in sediments
	Clutches, cemented	Groups of eggs (i.e. hundreds of eggs in one clutch) and cemented in sediments
	Clutches, free	Groups of eggs (i.e. hundreds of eggs in one clutch) and not cemented
	Clutches in vegetation	Groups of eggs (i.e. hundreds of eggs in one clutch) found in vegetation
	Clutches, terrestrial	Groups of eggs (i.e. hundreds of eggs in one clutch) can be aquatic but in some cases, in response to drying-up events, these clutches can be terrestrial or endophytic
Respiration	Tegument	Direct uptake of oxygen though the teguments
	Gills	The exchange of gas happens through the very thin walls of gill structures that contain hemolymph. Unlike in vertebrates, there are no red blood cells. Also referred as “blood gills”
	Plastron	A structure made up from projections of the cuticle that form a felt-like coat in which gaseous air is stocked
	Spiracle	Air enters through a series of external openings (spiracles). These external openings act as muscular valves in some insects. These lead to the internal respiratory system, structures with thin walls that branch out into tracheas also referred as “tracheal gills”.
Size	Size1	≤ 0.25 cm
	Size2	> 0.25–0.5 cm
	Size3	> 0.5–1 cm
	Size4	> 1–2 cm
	Size5	> 2–4 cm
	Size6	> 4–8 cm
	Size7	> 8 cm

Supplementary materials

Table S2. Trait allocation for each taxa in Ecuador (Ec) with trait classes of absorber (Ab), deposit feeder (DF), filter feeder (FF), parasite (Par), piercer (Pie), scraper (Scr) and shredder (Shr) for feeding strategy, burrower (Bur), crawler (Cra), flier (Fli), full water swimmer (FWS), interstitial endobenthic (IE), permanently attached (PA), surface swimmer (SS) and temporarily attached (TA) for locomotion, asexual reproduction (AS), clutches & cemented (CC), clutches & free (CF), clutches in vegetation (CV), clutches in terrestrial (CT), isolated eggs & clutches (IEC), isolated egss & free (IEF) and oviviparity for reproduction, gills (Gil), hydrostatic vesicle (HV), plastron (pla), spiracle (Spi) and tegument (Teg) for respiration, 1(≤0.25 cm), 2(>0.25–0.50 cm), 3 (>0.50–1.0 cm), 4 (>1.0–2.0 cm), 5 (>2.0–4.0 cm), 6 (>4.0–8.0 cm) and 7 (>8.0 cm) for maximal potential size. The values in the table are based on fuzzy coding obtained from a fuzzy-coded trait database (after Schmidt-Kloiber and Hering (2015), Tachet et al. (2000), Tomanova, Moya, and Oberdorff (2008)) while the trait classes encoded with “yes” were obtained from other trait database (after MarLIN (2006) and USEPA (2016), which obtained data from Poff et al. (2006), Vieira et al. (2006), EPA GCRP North Carolina (2010), EPA GCRP Maine (2010), EPA GCRP Utah (2010), Rankin and Yoder (2009), Huff, Hubler, Pan, and Drake (2008), Brandt (2001), Hubbard and Peters (1978)).

	Feeding style								Locomotion								Reproduction								Respiration					Maximal Potential Size						
Families	Ab	DF	FF	Par	Pre	Pie	Scr	Shr	Bur	Cra	Fli	FWS	IE	PA	SS	TA	As	CC	CF	CV	CT	IEC	IEF	Ovi	Gil	HV	Pla	Spi	Teg	1	2	3	4	5	6	7
Aeshnidae	0	0	0	0	3	0	0	0	0	5	0	0	0	0	0	0	0	0	0	3	3	0	2	0	3	0	0	0	1	0	0	0	0	2	3	0
Ampullariidae	0	0	0	0	0	0	yes	0	yes	0	0	0	0	0	0	yes	0	0	0	yes	0	yes	0	0	yes	0	0	0	0	0	0	0	yes	yes	0	0
Ancylidae	0	0	0	0	0	0	yes	0	yes	0	0	0	0	0	0	yes	0	0	0	yes	0	yes	0	0	yes	0	0	0	0	0	yes	yes	0	0	0	0
Baetidae	0	1	0	0	0	0	3	0	0	4	0	3	1	0	0	0	0	3	0	0	0	1	0	0	2	0	0	0	1	0	0	3	1	0	0	0
Belostomatidae	0	0	0	0	yes	yes	0	0	0	yes	0	yes	0	0	yes	0	0	0	0	yes	0	yes	0	yes	0	0	0	yes	0	0	0	0	yes	yes	0	0
Blephariceridae	0	0	0	0	0	0	3	0	0	2	0	0	0	0	0	2	0	3	0	0	0	0	0	0	3	0	0	0	1	0	0	3	0	0	0	0
Caenidae	0	3	0	0	0	0	0	1	1	5	0	0	1	0	0	0	0	0	0	0	0	2	1	0	3	0	0	0	1	0	2	3	0	0	0	0
Calamoceratidae	0	0	0	0	0	0	0	3	0	5	0	0	0	0	0	0	0	1	3	0	0	0	0	0	2	0	0	0	2	0	0	0	3	1	0	0
Calopterygidae	0	0	0	0	3	0	0	0	0	4	0	1	0	0	0	0	0	0	0	3	0	0	0	0	3	0	0	0	1	0	0	0	1	3	1	0
Cambaridae	0	0	0	0	3	0	0	3	3	1	0	0	0	0	0	0	0	3	0	0	0	0	0	3	3	0	0	0	0	0	0	0	0	0	0	3
Ceratopogonidae	0	1	0	0	3	0	0	1	3	1	0	3	0	0	1	0	0	3	0	0	0	1	0	0	3	0	0	0	0	0	0	1	3	0	0	0
Chaoboridae	0	0	0	0	3	0	0	0	0	1	0	5	0	0	1	0	0	0	3	0	0	0	1	0	0	1	0	0	3	0	0	0	3	0	0	0
Chironomidae	0	3	2	1	1	0	1	2	2	3	0	1	1	0	0	2	0	1	3	0	0	0	0	1	1	0	0	0	3	0	0	1	3	2	0	0
Chordodidae	0	0	0	yes	0	0	0	0	0	0	0	yes	0	0	0	0	0	0	0	yes	0	0	0	0	0	0	0	0	yes	0	0	0	0	0	0	yes
Coenagrionidae	0	0	0	0	3	0	0	0	0	5	0	0	0	0	0	0	0	0	0	3	0	0	0	0	3	0	0	0	1	0	0	0	3	0	0	0
Corbiculidae	0	0	3	0	0	0	0	0	4	1	0	1	0	0	0	0	0	0	0	0	0	0	1	3	3	0	0	0	1	0	0	0	0	1	3	0
Corixidae	0	0	0	0	0	3	2	3	0	2	0	3	0	0	0	0	0	2	0	0	0	3	0	0	0	0	1	1	1	3	1	0	0	0	0	0
Corydalidae	0	0	0	0	yes	0	0	0	yes	yes	0	yes	0	0	0	yes	0	0	0	yes	0	yes	0	0	yes	0	0	yes	yes	0	0	0	yes	yes	0	0
Coryphoridae	0	yes	0	0	0	0	yes	0	0	0	0	0	0	0	0	yes	0	0	0	0	0	yes	0	0	yes	0	0	0	0	0	yes	yes	0	0	0	0
Crambidae	0	0	0	0	0	yes	yes	10	0	6	0	2	0	0	0	yes	0	0	0	yes	0	0	0	0	yes	0	yes	yes	0	0	0	0	0	yes	0	0
Culicidae	0	2	0	0	2	0	1	0	0	0	0	0	0	0	5	0	0	0	3	0	1	0	1	0	0	0	0	3	0	0	1	3	0	0	0	0
Dixidae	0	0	3	0	1	0	0	1	0	1	0	1	0	0	3	1	0	3	1	0	1	0	0	0	0	0	0	3	0	0	0	3	0	0	0	0
Dryopidae	0	0	0	0	0	0	3	3	3	3	2	0	0	0	0	0	0	0	0	1	3	0	0	0	0	0	3	3	3	0	3	0	0	0	0	0
Dugesiidae	0	0	0	0	3	0	0	0	0	5	0	0	0	0	1	0	2	2	0	0	0	0	0	0	0	0	0	0	3	0	0	1	2	3	0	0
Dytiscidae	0	0	0	0	0	3	0	3	0	3	1	3	0	0	0	0	0	3	0	0	0	0	0	0	0	0	0	3	1	0	3	0	0	0	0	0
Elmidae	0	0	0	0	0	0	3	1	0	4	1	0	1	0	0	0	0	3	0	0	0	0	0	0	3	0	3	0	1	0	3	0	0	0	0	0
Empididae	0	0	0	0	3	0	0	0	1	2	0	0	1	0	0	0	0	0	0	0	3	0	0	0	0	0	0	1	1	0	0	3	0	0	0	0
Gerridae	0	0	0	0	1	3	0	0	0	0	3	0	0	0	4	0	0	2	0	0	3	1	0	0	0	0	0	3	0	0	0	1	3	0	0	0
Glossiphoniidae	0	0	0	1	0	3	0	0	0	3	0	0	0	0	0	1	0	0	1	0	0	0	0	2	0	0	0	0	3	0	0	0	3	3	0	0
Glossosomatidae	0	1	0	0	0	0	3	0	0	1	0	0	0	0	0	3	0	3	0	0	0	0	0	0	0	0	0	0	3	0	0	3	0	0	0	0
Gomphidae	0	0	0	0	3	0	0	0	4	1	0	0	0	0	0	0	0	0	0	0	0	3	1	0	3	0	0	0	1	0	0	0	0	3	0	0
Gyrinidae	0	0	0	0	0	3	0	3	0	3	1	3	0	0	3	0	0	3	0	0	0	0	0	0	3	0	0	3	1	0	2	3	0	0	0	0
Hebridae	0	0	0	0	yes	0	0	0	yes	yes	0	0	0	0	yes	0	0	0	0	yes	0	0	yes	0	0	0	yes	0	0	0	yes	yes	0	0	0	0
Helicopsychidae	0	0	0	0	0	0	3	1	1	4	0	0	0	0	0	1	0	2	0	0	0	2	0	0	0	0	0	0	3	0	0	3	0	0	0	0
Heteroceridae	0	yes	yes	0	0	0	yes	yes	0	0	0	0	0	0	yes	0	0	0	0	yes	0	yes	0	0	yes	0	0	yes	0	0	yes	yes	0	0	0	0
Hyallelidae	0	0	0	0	0	0	yes	0	0	0	0	yes	0	0	0	0	0	0	0	0	0	0	yes	0	yes	0	0	0	0	0	yes	yes	0	0	0	0
Hydrobiidae	0	0	0	0	0	0	3	0	1	3	0	0	0	0	0	0	0	0	0	0	0	3	0	0	3	0	0	0	1	0	3	0	0	0	0	0
Hydrobioscidae	0	0	0	0	yes	0	0	0	0	0	0	0	0	0	0	yes	0	yes	0	0	0	0	0	0	yes	0	0	0	yes	0	0	0	yes	yes	0	0
Hydrometridae	0	0	0	0	0	3	0	0	0	2	1	0	0	0	4	0	0	0	0	3	0	1	0	0	0	0	0	3	0	0	0	1	3	0	0	0
Hydrophilidae	0	0	0	0	1	0	0	3	0	3	1	3	0	0	0	0	0	0	3	0	0	0	0	0	0	0	2	3	1	0	0	0	0	2	3	0

Supplementary materials

	Feeding style								Locomotion								Reproduction								Respiration					Maximal Potential Size						
Families	Ab	DF	FF	Par	Pre	Pie	Scr	Shr	Bur	Cra	Fli	FWS	IE	PA	SS	TA	As	CC	CF	CV	CT	IEC	IEF	Ovi	Gil	HV	Pla	Spi	Teg	1	2	3	4	5	6	7
Hydropsychidae	0	0	3	0	1	0	0	0	0	2	0	0	0	0	0	3	0	3	0	0	0	0	0	0	3	0	0	0	2	0	0	1	3	1	0	0
Hydroptilidae	0	1	0	0	0	3	1	1	0	3	0	0	0	0	0	1	0	3	0	0	0	0	0	0	1	0	0	0	3	0	3	1	0	0	0	0
Lampyridae	0	0	0	0	0	yes	0	0	0	0	0	yes	0	0	0	0	0	yes	0	0	yes	0	0	0	0	0	0	yes	yes	yes	yes	yes	0	0	0	0
Leptoceridae	0	0	0	0	0	0	1	3	0	5	0	0	0	0	0	0	0	3	0	0	0	0	0	0	3	0	0	0	2	0	0	2	2	0	0	0
Leptohyphidae	0	yes	0	0	0	0	yes	0	0	0	0	0	0	0	0	yes	0	0	0	0	0	yes	0	0	yes	0	0	0	0	0	yes	yes	0	0	0	0
Leptophlebiidae	0	3	0	0	0	0	0	1	0	4	0	2	1	0	0	0	0	0	0	0	0	3	1	0	3	0	0	0	1	0	0	3	1	0	0	0
Libellulidae	0	0	0	0	3	0	0	0	1	4	0	2	0	0	0	0	0	0	0	0	0	3	2	0	3	0	0	0	1	0	0	0	0	3	0	0
Limoniidae	0	1	0	0	1	0	0	3	3	2	0	0	0	0	0	0	0	1	0	0	0	0	1	0	0	0	0	3	1	0	0	2	3	0	0	0
Lumbriculidae	1	3	0	0	0	0	0	0	0	0	0	0	1	0	0	0	3	2	0	0	0	0	0	0	0	0	0	0	3	0	0	0	0	3	3	3
Lymnaeidae	0	0	0	0	0	0	3	1	0	3	0	0	0	0	1	0	0	3	1	0	0	0	0	0	0	0	0	0	3	0	0	0	0	3	3	0
Macroveliidae	0	0	0	0	yes	yes	0	0	0	yes	0	0	0	0	0	0	0	0	0	0	0	yes	0	0	0	0	yes	0	0	0	yes	0	0	0	0	0
Megapodagrionidae	0	0	0	0	yes	0	0	0	yes	0	0	0	0	0	0	0	0	0	0	0	yes	0	0	0	yes	0	0	yes	0	0	0	yes	0	0	0	0
Mesoveliidae	0	0	0	0	0	3	0	0	0	1	1	0	0	0	4	0	0	0	0	3	0	1	0	0	0	0	0	3	0	0	3	0	0	0	0	0
Mysidae	0	yes	0	0	yes	0	0	0	yes	0	0	yes	0	0	0	0	0	0	0	0	0	0	yes	0	yes	0	0	0	0	0	yes	0	0	0	0	0
Naucoridae	0	0	0	0	1	3	0	0	0	0	1	3	0	0	2	0	0	0	0	3	0	1	0	0	0	0	0	3	0	0	0	0	3	0	0	0
Nereidae	0	yes	0	0	0	0	yes	0	yes	yes	0	yes	0	0	0	0	0	0	0	0	0	0	yes	0	0	0	0	0	yes	0	0	0	0	0	0	yes
Noteridae	0	0	0	0	3	0	0	3	3	0	1	3	0	0	0	0	0	3	0	0	0	0	0	0	0	0	0	3	3	0	3	0	0	0	0	0
Notonectidae	0	0	0	0	1	3	0	0	0	0	2	4	0	0	2	0	0	1	0	3	0	0	0	0	0	0	1	3	1	0	0	0	3	0	0	0
Ocypodidae	0	yes	0	0	0	0	0	0	yes	0	0	0	0	0	0	0	0	yes	0	0	0	0	0	0	yes	0	0	0	0	0	0	0	yes	0	0	0
Odontoceridae	0	0	0	0	3	0	0	3	2	3	0	0	0	0	0	0	0	0	0	0	3	0	0	0	3	0	0	0	2	0	0	0	3	1	0	0
Oligoneuriidae	0	2	3	0	0	0	2	0	0	4	0	1	0	0	0	0	0	0	0	0	0	1	3	0	3	0	0	0	1	0	0	0	3	0	0	0
Palaemonidae	0	0	0	0	yes	0	yes	0	0	yes	0	yes	0	0	0	0	0	yes	0	0	0	yes	0	yes	yes	0	0	0	0	0	0	0	yes	yes	0	0
Perlidae	0	0	0	0	1	0	0	3	0	5	0	0	0	0	0	0	0	0	2	0	0	3	0	0	3	0	0	0	0	0	0	0	0	3	0	0
Philopotamidae (Ec)	0	0	3	0	0	0	2	0	0	2	0	0	0	0	0	3	0	3	0	0	0	0	0	0	1	0	0	0	3	0	0	3	0	0	0	0
Philopotamidae (Phil)	0	0	3	0	0	0	2	0	0	2	0	0	0	0	0	3	0	3	0	0	0	0	0	0	1	0	0	0	3	0	0	3	0	0	0	0
Physidae	0	0	0	0	0	0	3	0	0	3	0	0	0	0	0	0	0	3	0	0	0	0	0	0	0	0	0	0	3	0	0	0	3	0	0	0
Planorbidae	0	0	0	0	0	0	2	1	0	4	0	0	0	0	2	0	0	3	0	0	0	0	0	0	2	0	0	0	3	0	0	0	3	0	0	0
Platystictidae	0	0	0	0	yes	0	0	0	1	3	0	1	0	0	0	0	0	0	0	0	yes	0	0	0	3	0	0	0	1	0	0	0	0	0	yes	0
Pleidae	0	0	0	0	0	3	0	0	0	0	0	5	0	0	0	0	0	0	0	3	0	0	0	0	0	0	0	3	0	1	3	0	0	0	0	0
Polycentropodidae	0	0	1	0	3	0	0	1	0	1	0	1	0	0	0	3	0	3	0	0	0	0	0	0	0	0	0	0	3	0	0	1	3	0	0	0
Polymitarcyidae	0	1	3	0	0	0	0	0	5	0	0	0	0	0	0	0	0	0	0	0	0	0	3	0	3	0	0	0	1	0	0	0	3	0	0	0
Psephenidae	0	0	0	0	0	0	3	1	0	5	0	0	0	0	0	0	0	yes	0	0	0	0	0	0	3	0	0	0	1	0	3	0	0	0	0	0
Ptilodactylidae	0	0	0	0	0	0	3	3	3	3	0	0	0	0	0	0	0	yes	0	0	0	0	0	0	3	0	0	0	0	0	2	2	3	0	0	0
Scirtidae	0	0	0	0	0	0	3	1	0	5	0	0	0	0	0	0	0	0	0	0	3	0	0	0	3	0	0	0	1	0	3	0	0	0	0	0
Simuliidae	0	0	3	0	0	0	1	0	0	2	0	0	1	0	0	4	0	3	1	0	1	0	1	0	1	0	0	3	3	0	2	3	0	0	0	0
Sphaeriidae	0	0	3	0	0	0	0	0	4	1	0	0	0	0	0	2	0	0	0	0	0	0	0	3	3	0	0	0	1	0	0	0	3	1	0	0
Staphylinidae	2	2	0	0	1	0	1	2	1	3	0	0	0	0	0	0	0	0	0	yes	0	0	0	0	0	0	0	0	1	3	0	0	0	0	0	0
Stratiomyidae	0	2	0	0	1	0	1	3	0	3	0	0	0	0	3	0	0	2	0	0	1	0	3	0	0	0	0	3	0	0	0	2	2	1	1	0
Tabanidae	0	0	0	0	0	3	0	1	5	2	0	0	0	0	1	0	0	3	0	0	2	0	0	0	0	0	0	3	0	0	0	0	0	3	1	0
Thiaridae	0	0	0	0	0	0	yes	0	yes	0	0	0	0	0	0	yes	0	yes	0	0	0	yes	0	0	yes	0	0	yes	0	0	0	0	yes	0	0	0
Trichodactylidae	0	0	0	0	yes	0	0	0	yes	0	0	0	0	0	0	0	0	0	0	0	0	0	0	yes	yes	0	0	0	0	0	0	0	0	yes	0	0
Tubificidae	1	3	0	0	0	0	0	0	3	0	0	0	1	0	0	2	2	3	0	0	0	0	0	0	0	0	0	0	3	0	0	0	0	0	3	0
Veliidae	0	0	0	0	1	3	0	0	0	0	1	0	0	0	4	0	0	3	0	0	3	0	0	0	0	0	0	3	0	0	0	3	0	0	0	0

Supplementary materials

Table S3. Estimates and *p*-values of each feeding strategy. For all numeric variables, the model containing both the linear and quadratic terms is based on the mean-centered variables as described in the text. The final models are explained in the text and are highlighted in yellow.

	Deposit Feeeder		Filter feeder		Parasite		Piercer		Predator		Scraper		Shredder	
	Estimate	p-values	Estimate	p-values	Estimate	p-values	Estimate	p-values	Estimate	p-values	Estimate	p-values	Estimate	p-values
(Intercept)	3.03E+00	**4.29E-63**	2.49E+00	8.01E-24	1.19E-01	5.84E-01	2.13E+00	**8.95E-37**	2.73E+00	7.36E-60	3.76E+00	**1.06E-98**	2.31E+00	6.83E-42
I(ElevationT^2)	-6.17E-06	**6.97E-03**	-2.17E-06	4.87E-01	1.73E-06	5.21E-01	-4.63E-06	**3.11E-02**	-1.19E-06	5.74E-01	-5.33E-06	**1.82E-02**	-4.60E-07	8.30E-01
ElevationT	5.73E-03	**3.53E-05**	2.99E-03	1.13E-01	-1.22E-03	4.61E-01	3.65E-03	**4.80E-03**	1.44E-03	2.59E-01	4.61E-03	**7.36E-04**	2.21E-03	8.97E-02
(Intercept)2					2.17E-01	2.28E-01					3.45E+00	2.48E-106	2.13E+00	3.81E-48
I(Elevation^2)					-4.76E-08	9.55E-01					1.90E-06	8.60E-03	2.27E-06	5.49E-04
(Intercept)1			2.05E+00	**5.40E-17**	2.51E-01	2.33E-01			2.52E+00	1.93E-52	3.18E+00	1.11E-69	1.96E+00	**7.32E-31**
Elevation			2.02E-03	**2.11E-02**	-2.33E-04	7.64E-01			9.16E-04	1.21E-01	2.20E-03	6.58E-04	2.00E-03	**8.15E-04**
(Intercept)3	2.71E+00	7.86E-60	2.49E+00	1.35E-27	2.91E-01	1.39E-01	1.68E+00	**1.34E-28**	2.52E+00	**5.48E-61**	3.36E+00	5.43E-89	2.30E+00	1.46E-47
I(VelocityT^2)	1.71E-01	8.66E-01	-1.01E+00	4.70E-01	-1.03E+00	4.47E-01	2.16E+00	1.65E-02	1.67E+00	7.09E-02	2.22E+00	2.99E-02	1.22E-01	8.99E-01
VelocityT	2.49E+00	5.78E-05	1.87E+00	2.82E-02	2.50E-01	7.32E-01	7.13E-01	2.04E-01	9.96E-02	8.60E-01	4.42E-01	4.78E-01	1.36E+00	2.20E-02
(Intercept)5	2.24E+00	6.40E-40	2.22E+00	8.16E-22	2.54E-01	1.95E-01	1.58E+00	**2.98E-25**	2.54E+00	1.03E-61	3.32E+00	**5.11E-87**	2.10E+00	4.33E-39
I(Velocity^2)	2.62E+00	1.40E-09	1.13E+00	5.78E-02	-2.18E-01	6.85E-01	1.46E+00	**1.13E-04**	6.70E-01	8.83E-02	1.20E+00	**5.85E-03**	1.16E+00	4.36E-03
(Intercept)4	1.88E+00	**1.40E-17**	1.92E+00	**1.93E-10**	2.21E-01	3.89E-01	1.44E+00	1.46E-12	2.52E+00	1.16E-35	3.24E+00	9.08E-48	1.85E+00	**1.68E-18**
Velocity	2.53E+00	**2.46E-07**	1.48E+00	**2.85E-02**	-2.24E-02	9.69E-01	1.33E+00	3.05E-03	4.74E-01	2.98E-01	1.03E+00	4.04E-02	1.41E+00	**2.66E-03**
(Intercept)21	3.21E+00	1.18E-56	2.69E+00	6.13E-27	6.75E-01	**1.75E-02**	1.95E+00	2.18E-34	2.69E+00	1.22E-72	3.71E+00	1.18E-107	2.54E+00	3.71E-55
I(TurbidityT^2)	-1.28E-02	4.03E-02	-1.18E-02	1.01E-01	-6.70E-02	**2.71E-02**	-8.24E-04	7.27E-01	-1.54E-03	4.55E-01	-3.37E-03	1.33E-01	-4.60E-03	4.17E-02
TurbidityT	2.13E-02	7.27E-01	3.90E-02	6.07E-01	-5.68E-02	4.31E-01	-6.56E-02	1.83E-01	-3.79E-02	4.07E-01	-3.22E-03	9.50E-01	4.95E-02	3.18E-01
(Intercept)22	3.19E+00	**7.53E-79**	2.65E+00	**2.00E-36**	4.17E-01	2.66E-02	2.07E+00	1.59E-47	2.76E+00	3.77E-93	3.73E+00	**8.19E-132**	2.49E+00	**5.93E-63**
I(Turbidity^2)	-6.40E-03	**6.45E-03**	-5.45E-03	**3.00E-02**	-1.13E-02	9.27E-02	-2.69E-03	1.43E-02	-2.27E-03	5.79E-03	-2.66E-03	**1.69E-03**	-1.92E-03	**2.55E-02**
(Intercept)23	3.39E+00	9.42E-66	2.82E+00	1.25E-30	5.13E-01	1.75E-02	2.26E+00	**2.61E-40**	2.93E+00	**5.87E-76**	3.89E+00	8.04E-104	2.51E+00	1.26E-46
Turbidity	-9.92E-02	7.62E-04	-8.57E-02	1.60E-02	-9.19E-02	4.62E-02	-7.91E-02	**3.83E-03**	-6.81E-02	**3.40E-03**	-6.59E-02	7.86E-03	-2.23E-02	3.50E-01

Supplementary materials

Table S4. Estimates and *p*-values of each respiration mode. For all numeric variables, the model containing both the linear and quadratic terms is based on the mean-centered variables as described in the text. The final models are explained in the text and are highlighted in yellow.

	Gill		Plastron		Spiracle		Tegument	
	Estimate	p-values	Estimate	p-values	Estimate	p-values	Estimate	p-values
(Intercept)	4.08E+00	**1.09E-148**	1.10E+00	4.86E-06	2.97E+00	**3.96E-53**	3.43E+00	**8.75E-98**
I(ElevationT^2)	-5.32E-06	**7.42E-03**	-5.01E-06	1.01E-01	-4.43E-06	7.27E-02	-1.33E-06	5.19E-01
ElevationT	5.26E-03	**1.23E-05**	5.93E-03	1.57E-03	2.56E-03	8.46E-02	1.70E-03	1.73E-01
(Intercept)1	3.36E+00	3.62E-97	2.05E-01	4.21E-01	2.69E+00	2.68E-44	3.29E+00	1.67E-124
Elevation	3.08E-03	9.15E-08	4.16E-03	1.27E-06	5.09E-04	4.64E-01	1.09E-06	8.63E-02
(Intercept)2	3.72E+00	4.51E-147	5.93E-01	**8.98E-03**	2.77E+00	4.26E-63	3.19E+00	1.01E-86
I(Elevation^2)	3.03E-06	4.79E-06	5.35E-06	**5.55E-08**	1.67E-07	8.27E-01	1.09E-03	6.04E-02
(Intercept)3	3.78E+00	5.74E-139	1.08E+00	5.29E-06	2.42E+00	**1.13E-43**	3.37E+00	**1.26E-111**
I(VelocityT^2)	1.26E+00	1.69E-01	-1.45E+00	3.15E-01	3.43E+00	**1.12E-03**	-5.54E-02	9.52E-01
VelocityT	1.53E+00	6.15E-03	3.46E+00	1.62E-04	-7.68E-01	2.32E-01	8.38E-01	1.32E-01
(Intercept)5	3.50E+00	7.05E-120	4.09E-01	1.06E-01	2.66E+00	1.52E-50	3.09E+00	8.71E-56
I(Velocity^2)	1.97E+00	4.47E-07	3.35E+00	8.48E-08	5.35E-01	2.44E-01	8.20E-01	6.43E-02
(Intercept)4	3.29E+00	**1.28E-61**	-9.40E-02	7.75E-01	2.70E+00	1.22E-30	3.24E+00	5.97E-103
Velocity	1.83E+00	**4.42E-05**	3.20E+00	**6.45E-06**	2.28E-01	6.67E-01	6.90E-01	7.58E-02
(Intercept)21	4.17E+00	2.20E-150	1.13E+00	2.51E-05	2.73E+00	4.90E-56	3.52E+00	1.21E-130
I(TurbidityT^2)	-4.18E-03	5.10E-02	1.52E-03	7.11E-01	-4.06E-04	8.61E-01	-6.21E-03	8.71E-03
TurbidityT	5.70E-03	9.07E-01	-1.53E-01	6.57E-02	-7.08E-02	1.81E-01	3.16E-02	4.75E-01
(Intercept)22	4.18E+00	**7.99E-183**	1.48E+00	1.51E-09	2.85E+00	4.81E-74	3.50E+00	**3.72E-157**
I(Turbidity^2)	-3.01E-03	**2.03E-04**	-5.08E-03	1.21E-01	-2.37E-03	9.82E-03	-3.57E-03	**1.87E-04**
(Intercept)23	4.34E+00	2.86E-141	1.73E+00	**3.63E-09**	3.04E+00	**5.79E-62**	3.65E+00	6.63E-120
Turbidity	-6.87E-02	3.49E-03	-1.34E-01	**1.77E-02**	-7.90E-02	**3.22E-03**	-6.77E-02	2.14E-03

Supplementary materials

Table S5. Estimates and *p*-values of each locomotion mode. For all numeric variables, the model containing both the linear and quadratic terms is based on the mean-centered variables as described in the text. The final models are explained in the text and are highlighted in yellow.

	Burrower		Crawler		Flier		Fullwaterswimmer		InterstitialEndobenthic		Surfaceswimmer		Temporarily attached	
	Estimate	p-values	Estimate	p-values	Estimate	p-values	Estimate	p-values	Estimate	p-values	Estimate	p-values	Estimate	p-values
(Intercept)	2.49E+00	**9.07E-44**	3.46E+00	1.27E-107	7.53E-01	**1.43E-04**	2.31E+00	**4.80E-46**	1.16E+00	5.81E-10	1.89E+00	**1.42E-18**	3.71E+00	**3.23E-97**
I(ElevationT^2)	2.08E-06	3.58E-01	-1.44E-06	4.67E-01	-7.56E-06	**3.55E-03**	-6.90E-07	7.36E-01	-1.38E-06	5.57E-01	-6.96E-06	**1.37E-02**	-8.05E-06	**3.50E-04**
ElevationT	-1.12E-03	4.13E-01	2.71E-03	2.43E-02	6.31E-03	**6.02E-05**	9.41E-04	4.48E-01	2.42E-03	9.22E-02	4.21E-03	**1.09E-02**	6.84E-03	**5.18E-07**
(Intercept)2	2.58E+00	1.23E-65	3.24E+00	2.49E-127	3.14E-01	1.12E-01	2.23E+00	2.12E-59	9.62E-01	**5.17E-09**	1.55E+00	1.67E-16	3.32E+00	1.63E-87
I(Elevation^2)	1.96E-07	7.79E-01	2.30E-06	1.93E-04	3.14E-06	1.22E-04	6.31E-07	3.13E-01	2.04E-06	**3.94E-03**	1.03E-06	2.27E-01	3.14E-06	4.61E-05
(Intercept)1	2.59E+00	1.28E-48	3.05E+00	**1.26E-85**	-4.28E-02	8.48E-01	2.17E+00	7.54E-42	7.94E-01	2.96E-05	1.37E+00	3.10E-10	2.83E+00	3.50E-52
Elevation	6.05E-05	9.24E-01	2.06E-03	**2.07E-04**	3.08E-03	1.66E-05	6.30E-04	2.71E-01	1.83E-03	4.67E-03	1.39E-03	7.06E-02	3.65E-03	4.00E-08
(Intercept)3	2.44E+00	**3.45E-51**	3.38E+00	4.18E-116	3.31E-01	9.33E-02	2.17E+00	**3.23E-48**	1.17E+00	3.13E-11	1.19E+00	5.41E-10	3.34E+00	2.07E-84
I(VelocityT^2)	1.47E+00	1.33E-01	4.92E-01	5.84E-01	1.54E+00	1.78E-01	1.20E+00	1.82E-01	-5.58E-01	6.01E-01	3.39E+00	2.86E-03	9.95E-01	3.41E-01
VelocityT	-1.17E+00	**5.00E-02**	1.15E+00	3.59E-02	1.57E+00	3.72E-02	-3.01E-02	9.56E-01	1.39E+00	3.74E-02	2.87E-01	6.84E-01	1.87E+00	3.22E-03
(Intercept)5	2.61E+00	5.12E-57	3.19E+00	4.53E-103	5.96E-02	7.70E-01	2.19E+00	2.45E-49	9.32E-01	2.67E-07	1.21E+00	**3.23E-10**	2.99E+00	4.18E-68
I(Velocity^2)	-5.46E-02	8.98E-01	1.23E+00	1.22E-03	2.07E+00	9.10E-06	4.18E-01	2.72E-01	1.10E+00	1.30E-02	1.45E+00	**2.71E-03**	2.21E+00	6.06E-07
(Intercept)4	2.72E+00	4.25E-37	2.99E+00	**9.45E-54**	-1.96E-01	4.74E-01	2.19E+00	4.45E-29	7.09E-01	**2.83E-03**	1.15E+00	9.88E-06	2.72E+00	**1.93E-33**
Velocity	-3.96E-01	4.16E-01	1.30E+00	**2.88E-03**	1.99E+00	**5.09E-04**	2.72E-01	5.38E-01	1.25E+00	**1.54E-02**	1.18E+00	3.97E-02	2.12E+00	**2.83E-05**
(Intercept)21	2.69E+00	4.47E-65	3.59E+00	9.39E-128	5.90E-01	1.11E-02	2.28E+00	1.80E-56	1.24E+00	9.07E-08	1.63E+00	2.43E-13		
I(TurbidityT^2)	-4.07E-03	7.17E-02	-3.52E-03	8.03E-02	-1.34E-03	8.05E-01	-1.53E-03	4.59E-01	-1.38E-02	3.55E-01	-5.99E-03	4.29E-01		
TurbidityT	2.07E-02	6.69E-01	6.65E-03	8.84E-01	-1.06E-01	1.41E-01	-3.95E-02	3.73E-01	-7.97E-02	1.90E-01	-8.04E-02	2.28E-01		
(Intercept)22	2.68E+00	**5.51E-78**	3.60E+00	**5.95E-155**	8.50E-01	4.01E-05	2.35E+00	9.54E-74	1.39E+00	1.16E-15	1.81E+00	5.12E-22	3.78E+00	7.09E-117
I(Turbidity^2)	-2.47E-03	**6.37E-03**	-2.45E-03	**1.26E-03**	-7.13E-03	1.30E-01	-2.33E-03	8.02E-03	-1.07E-02	3.88E-02	-8.11E-03	4.20E-02	-3.93E-03	4.50E-04
(Intercept)23	2.80E+00	1.79E-61	3.72E+00	5.29E-119	1.04E+00	**2.21E-05**	2.52E+00	**1.12E-60**	1.57E+00	**8.41E-15**	2.00E+00	**5.99E-20**	3.97E+00	**2.47E-92**
Turbidity	-5.50E-02	2.25E-02	-5.34E-02	1.55E-02	-1.15E-01	**2.43E-02**	-6.91E-02	**3.34E-03**	-1.23E-01	**4.00E-03**	-1.17E-01	**4.19E-03**	-8.21E-02	**2.52E-03**

Supplementary materials

Table S6. Estimates and *p*-values of each reproduction mode. For all numeric variables, the model containing both the linear and quadratic terms is based on the mean-centered variables as described in the text. The final models are explained in the text and are highlighted in yellow.

	ClutchesCemented		ClutchesFree		ClutchesVegetation		ClutchesTerrestrial		Isolatedeggscemented		IsolatedeggsFree		Oviviparity	
	Estimate	p-values	Estimate	p-values	Estimate	p-values	Estimate	p-values	Estimate	p-values	Estimate	p-values	Estimate	p-values
(Intercept)	3.68E+00	2.04E-96	1.91E+00	**1.53E-24**	1.70E+00	6.23E-27	1.33E+00	**1.40E-10**	3.79E+00	**1.68E-108**	1.55E+00	**2.33E-14**	1.10E+00	**7.92E-08**
I(ElevationT^2)	-2.91E-06	1.92E-01	3.08E-06	1.88E-01	-2.88E-06	1.53E-01	-5.45E-06	**4.36E-02**	-7.02E-06	**1.20E-03**	-6.66E-07	7.95E-01	1.75E-06	5.00E-01
ElevationT	3.57E-03	8.15E-03	-2.01E-03	1.58E-01	2.63E-03	3.07E-02	3.67E-03	**2.15E-02**	5.93E-03	**6.29E-06**	1.17E-03	4.51E-01	-1.64E-03	2.94E-01
(Intercept)2	3.40E+00	2.72E-110	2.08E+00	8.46E-40	1.51E+00	2.00E-27	1.05E+00	8.48E-09	3.43E+00	3.67E-102	1.45E+00	4.42E-17	1.23E+00	5.28E-13
I(Elevation^2)	2.40E-06	5.88E-04	3.97E-08	9.57E-01	1.07E-06	8.26E-02	9.42E-07	2.47E-01	2.77E-06	1.61E-04	9.52E-07	2.22E-01	-4.37E-07	5.92E-01
(Intercept)1	3.17E+00	**2.96E-73**	2.13E+00	8.66E-31	1.37E+00	**1.58E-17**	8.93E-01	2.44E-05	2.99E+00	1.32E-63	1.37E+00	9.79E-12	1.30E+00	7.17E-11
Elevation	2.25E-03	**3.55E-04**	-2.58E-04	7.00E-01	1.23E-03	**2.78E-02**	1.25E-03	8.89E-02	3.24E-03	3.77E-07	8.86E-04	2.15E-01	-6.43E-04	3.88E-01
(Intercept)3	3.52E+00	3.30E-99	2.06E+00	**3.00E-33**	1.49E+00	6.68E-24	7.59E-01	2.57E-05	3.35E+00	**3.10E-93**	1.38E+00	**2.78E-13**	1.29E+00	**6.29E-12**
I(VelocityT^2)	8.18E-01	4.21E-01	1.21E-01	9.09E-01	9.55E-01	2.75E-01	2.24E+00	3.30E-02	1.97E+00	**4.79E-02**	1.74E+00	1.21E-01	-1.18E+00	3.44E-01
VelocityT	9.56E-01	1.22E-01	-6.84E-01	2.79E-01	5.38E-01	3.27E-01	1.12E+00	9.62E-02	1.44E+00	**1.73E-02**	-3.75E-01	5.89E-01	8.11E-02	9.06E-01
(Intercept)5	3.36E+00	5.43E-91	2.18E+00	3.74E-37	1.40E+00	**3.37E-21**	5.83E-01	**1.43E-03**	3.10E+00	1.32E-80	1.50E+00	2.00E-15	1.29E+00	5.25E-12
I(Velocity^2)	1.18E+00	6.19E-03	-5.18E-01	2.61E-01	8.67E-01	**1.80E-02**	1.86E+00	**1.79E-05**	2.10E+00	5.83E-07	2.16E-01	6.56E-01	-4.57E-01	3.74E-01
(Intercept)4	3.18E+00	**6.08E-48**	2.29E+00	1.02E-24	1.30E+00	4.19E-11	4.20E-01	8.70E-02	2.92E+00	1.66E-41	1.55E+00	3.74E-10	1.29E+00	1.09E-07
Velocity	1.25E+00	**1.13E-02**	-6.48E-01	2.04E-01	8.39E-01	5.31E-02	1.65E+00	1.81E-03	1.84E+00	1.56E-04	-2.59E-02	9.63E-01	-2.76E-01	6.20E-01
(Intercept)21	3.78E+00	1.50E-113	2.61E+00	6.44E-28	1.66E+00	**5.33E-29**	6.88E-01	6.44E-04	3.78E+00	2.24E-107	1.45E+00	4.18E-15	1.33E+00	**1.17E-13**
I(TurbidityT^2)	-4.08E-03	6.69E-02	-6.96E-02	1.06E-03	-2.11E-03	3.14E-01	4.75E-03	1.47E-01	-4.03E-03	9.34E-02	6.53E-05	9.81E-01	-4.81E-03	7.68E-02
TurbidityT	1.63E-02	7.49E-01	-1.11E-02	8.34E-01	1.37E-03	9.76E-01	-2.33E-01	2.64E-04	-1.26E-02	8.11E-01	-8.87E-02	1.22E-01	6.17E-02	2.61E-01
(Intercept)22	3.78E+00	**1.41E-136**	2.31E+00	**2.34E-47**	1.67E+00	2.24E-35			3.82E+00	**3.48E-133**	1.61E+00	1.54E-22	1.27E+00	1.30E-14
I(Turbidity^2)	-2.61E-03	**1.74E-03**	-1.25E-02	**7.48E-03**	-1.54E-03	6.69E-02			-3.42E-03	**3.52E-04**	-2.63E-03	4.31E-02	-1.77E-03	1.04E-01
(Intercept)23	3.89E+00	1.11E-104	2.48E+00	1.41E-41	1.75E+00	2.38E-28	1.61E+00	**4.34E-13**	4.04E+00	6.80E-107	1.82E+00	**3.02E-20**	1.33E+00	7.45E-12
Turbidity	-5.23E-02	3.22E-02	-1.18E-01	4.75E-04	-3.66E-02	1.13E-01	-1.91E-01	**8.23E-04**	-8.84E-02	6.48E-04	-8.75E-02	**9.54E-03**	-3.19E-02	2.52E-01

Supplementary materials

Table S7. Estimates and *p*-values of each size category. For all numeric variables, the model containing both the linear and quadratic terms is based on the mean-centered variables as described in the text. The final models are explained in the text and are highlighted in yellow.

	Size1		Size2		Size3		Size4		Size5		Size6		Size7	
	Estimate	p-values	Estimate	p-values	Estimate	p-values	Estimate	p-values	Estimate	p-values	Estimate	p-values	Estimate	p-values
(Intercept)	9.31E-01	**1.29E-02**	2.71E+00	**2.70E-58**	3.56E+00	**1.48E-90**	3.59E+00	**1.53E-102**	2.63E+00	3.12E-52	9.82E-01	**1.45E-05**	-3.29E+00	**3.45E-05**
I(ElevationT^2)	-6.18E-06	2.40E-01	-7.63E-06	**3.66E-04**	-5.40E-06	**1.56E-02**	-2.40E-06	2.55E-01	8.06E-07	7.11E-01	1.22E-06	6.77E-01	8.41E-06	3.17E-01
ElevationT	1.86E-03	5.19E-01	7.90E-03	**1.45E-09**	4.79E-03	**3.89E-04**	2.03E-03	1.12E-01	8.51E-04	5.19E-01	-2.27E-03	1.90E-01	-3.85E-03	4.95E-01
(Intercept)2	8.04E-01	1.02E-02	2.19E+00	1.20E-37	3.24E+00	4.22E-93	3.44E+00	1.30E-129	2.55E+00	1.11E-68	1.19E+00	1.80E-10	-2.89E+00	8.53E-07
I(Elevation^2)	-1.60E-06	3.11E-01	5.98E-06	1.13E-14	2.33E-06	1.33E-03	6.50E-07	3.21E-01	1.49E-06	2.52E-02	-1.76E-06	8.59E-02	1.16E-06	5.79E-01
(Intercept)1	7.50E-01	4.18E-02	1.57E+00	5.24E-18	2.92E+00	1.44E-59	3.35E+00	5.81E-91	2.47E+00	**2.16E-47**	1.32E+00	1.49E-09	-2.90E+00	1.95E-05
Elevation	-2.74E-04	8.38E-01	5.23E-03	2.05E-16	2.58E-03	5.89E-05	8.15E-04	1.71E-01	1.21E-03	**4.61E-02**	-1.68E-03	5.59E-02	6.75E-04	7.56E-01
(Intercept)3	8.44E-01	**1.06E-02**	2.40E+00	5.03E-47	3.25E+00	9.30E-91	3.36E+00	**1.15E-105**	2.58E+00	**5.67E-58**	1.20E+00	**1.25E-08**	-2.59E+00	**2.66E-04**
I(VelocityT^2)	-3.53E+00	1.06E-01	5.27E-01	6.04E-01	3.76E-01	7.02E-01	1.40E+00	1.36E-01	1.29E+00	1.86E-01	-1.56E+00	3.02E-01	-3.00E+00	7.16E-01
VelocityT	2.47E+00	**4.68E-02**	3.21E+00	3.47E-07	1.87E+00	1.79E-03	-3.41E-01	5.49E-01	1.35E-01	8.21E-01	-2.21E-01	7.71E-01	-1.02E-01	9.66E-01
(Intercept)5	3.92E-01	2.48E-01	1.78E+00	8.05E-25	2.92E+00	1.66E-72	3.43E+00	1.71E-109	2.58E+00	5.60E-58	1.24E+00	2.10E-09	-2.63E+00	4.22E-05
I(Velocity^2)	1.36E+00	1.13E-01	3.66E+00	5.96E-17	1.94E+00	3.54E-06	3.13E-01	4.34E-01	5.96E-01	1.50E-01	-8.63E-01	1.59E-01	-8.49E-01	7.22E-01
(Intercept)4	7.40E-03	9.87E-01	1.35E+00	**1.86E-09**	2.63E+00	**2.28E-35**	3.45E+00	1.00E-64	2.56E+00	1.65E-33	1.31E+00	1.17E-06	-2.63E+00	1.22E-03
Velocity	1.79E+00	6.81E-02	3.30E+00	**2.44E-11**	1.97E+00	**3.37E-05**	1.47E-01	7.49E-01	4.45E-01	3.51E-01	-6.64E-01	2.86E-01	-4.64E-01	8.14E-01
(Intercept)21			3.12E+00	2.52E-52	3.58E+00	5.73E-99	3.54E+00	2.48E-125	2.73E+00	2.36E-68	1.67E+00	**2.91E-09**	-2.60E+00	**5.13E-05**
I(TurbidityT^2)			-4.57E-03	1.15E-01	-5.52E-03	3.38E-02	-2.56E-03	1.95E-01	-2.85E-03	2.42E-01	-5.34E-02	**2.57E-02**	-7.68E-03	7.08E-01
TurbidityT			8.94E-03	8.86E-01	1.23E-02	8.13E-01	-1.03E-02	8.21E-01	-3.97E-02	4.07E-01	1.59E-01	**1.24E-02**	6.37E-02	7.48E-01
(Intercept)22	8.02E-01	8.60E-03	3.13E+00	**1.23E-63**	3.59E+00	**3.63E-121**	3.57E+00	**2.09E-154**	2.80E+00	**4.91E-89**	1.22E+00	4.58E-11	-2.71E+00	1.33E-06
I(Turbidity^2)	-4.25E-03	2.30E-01	-3.12E-03	**6.12E-03**	-3.74E-03	**4.61E-04**	-2.24E-03	**2.42E-03**	-3.35E-03	**2.34E-03**	-3.96E-03	8.79E-02	-1.80E-03	7.53E-01
(Intercept)23	1.05E+00	**4.10E-03**	3.25E+00	3.62E-50	3.77E+00	2.09E-95	3.71E+00	1.07E-121	3.01E+00	3.95E-73	1.17E+00	5.38E-08	-2.71E+00	3.69E-05
Turbidity	-1.10E-01	**9.21E-02**	-5.98E-02	4.68E-02	-7.86E-02	2.14E-03	-6.10E-02	5.58E-03	-8.79E-02	5.88E-04	-1.67E-02	5.73E-01	-1.70E-02	8.64E-01

Supplementary materials

Table S8. Estimated tipping points with their standard errors in parenthesis of each feeding strategy trait with respect to elevation, velocity and turbidity. ↓ indicates decrease after tipping point, ↑ indicate increase after the tipping point and – indicates a constant after the tipping point. * significant at 0.05 level, ***significant at 0.001 level

	Turbidity	Elevation
**Traits**		
Tegument	5.1 (±1.6)↓*	
**Diversity**		
Shannon–Weaver	18.4 (±3.4)↑*	17.5(±4.4)↓***
Simpson		6 (±2.5) –*
Pielou's evenness	18.4 (±3.1)↑***	
Richness		22 (±5.9)↑ ***

References

Brandt, D. (2001). Temperature Preferences and Tolerances for 137 Common Idaho Macroinvertebrate Taxa.

EPA GCRP Maine. (2010). EPA GCRP State Biomonitoring Data Climate Change Pilot Project 2010: Freshwater Biological Traits Table for Maine.

EPA GCRP North Carolina. (2010). EPA GCRP State Biomonitoring Data Climate Change Pilot Project 2010: Freshwater Biological Traits Table for North Carolina

EPA GCRP Utah. (2010). EPA GCRP State Biomonitoring Data Climate Change Pilot Project 2010: Freshwater Biological Traits Table for Utah.

Hubbard, M. D., & Peters, W. L. (1978). Environmental Requirements and Pollution Tolerance of Ephemeroptera. Report No. EPA-600/4-78-061.

Huff, D. D., Hubler, S. L., Pan, Y., & Drake, D. L. (2008). Detecting Shifts in Macroinvertebrate Community Requirements: Implicating Causes of Impairment in Streams.

MarLIN. (2006). BIOTIC - Biological Traits Information Catalogue. Marine Life Information Network. Plymouth: Marine Biological Association of the United Kingdom. Retrieved from www.marlin.ac.uk/biotic

Poff, N. L., Olden, J. D., Vieira, N. K. M., Finn, D. S., Simmons, M. P., & Kondratieff, B. C. (2006). Functional trait niches of North American lotic insects: traits-based ecological applications in light of phylogenetic relationships. JOURNAL OF THE NORTH AMERICAN BENTHOLOGICAL SOCIETY, 25(4), 730-755. doi:Doi 10.1899/0887-3593(2006)025[0730:Ftnona]2.0.Co;2

Rankin, E. T., & Yoder, C. O. (2009). Temporal Change in Regional Reference Condition as a Potential Indicator of Global Climate Change: Analysis of the Ohio Regional Reference Condition Database (1980-2006).

Schmidt-Kloiber, A., & Hering, D. (2015). www.freshwaterecology.info - An online tool that unifies, standardises and codifies more than 20,000 European freshwater organisms and their ecological preferences. Ecological Indicators, 53, 271-282. doi:10.1016/j.ecolind.2015.02.007

Tachet, H., Richoux, P., Bournaud, M., & Usseglio-Polatera, P. (2000). Invertêbrés d'Eau Douce:Systématique, Biologie, Écologie. Paris, France: CNRS éditions.

Tomanova, S., Moya, N., & Oberdorff, T. (2008). Using Macroinvertebrate Biological Traits for Assessing Biotic Integrity of Neotropical Streams. River Research and Applications, 24(9), 1230-1239. doi:10.1002/rra.1148

USEPA. (2016). Traits - Data Sources and Metadata. Retrieved from https://www.epa.gov/risk/traits-data-sources-and-metadata

Supplementary materials

Vieira, N. K. M., Poff, N. L., Carlisle, D. M., Moulton II, S. R., Koski, M. K., & Kondratieff, B. C. (2006). A Database of Lotic Invertebrate Traits for North America: : U.S. Geological Survey Data Series 18. http://pubs.water.usgs.gov/ds18. Retrieved Oct. 15, 2015
